# Autologous Skin Fibroblast‐Based PLGA Nanoparticles for Treating Multiorgan Fibrosis

**DOI:** 10.1002/advs.202200856

**Published:** 2022-05-23

**Authors:** Qiang Long, Zehua Liu, Qianwen Shao, Hongpeng Shi, Shixing Huang, Chenyu Jiang, Bei Qian, Yiming Zhong, Xiaojun He, Xiaogang Xiang, Yang Yang, Bing Li, Xiaoxiang Yan, Qiang Zhao, Xiaoli Wei, Hélder A. Santos, Xiaofeng Ye

**Affiliations:** ^1^ Department of Cardiovascular Surgery Ruijin Hospital Shanghai Jiao Tong University School of Medicine Shanghai 200025 China; ^2^ Department of Biomedical Engineering, W.J. Kolff Institute for Biomedical Engineering and Materials Science University Medical Center Groningen/University of Groningen Ant. Deusinglaan 1 Groningen 9713 AV The Netherlands; ^3^ Drug Research Program Division of Pharmaceutical Chemistry and Technology Faculty of Pharmacy University of Helsinki Helsinki FI‐00014 Finland; ^4^ Department of Pharmacology School of Basic Medical Sciences Fudan University Shanghai 200032 China; ^5^ Department of Infectious Diseases Ruijin Hospital Shanghai Jiao Tong University School of Medicine Shanghai 200025 China; ^6^ Department of Thoracic Surgery Shanghai Pulmonary Hospital School of Medicine Tongji University Shanghai 200000 China; ^7^ Department of Respiratory and Critical Care Medicine Shanghai Pulmonary Hospital School of Medicine Tongji University Shanghai 200000 China; ^8^ Department of Cardiovascular Medicine Ruijin Hospital Shanghai Jiao Tong University School of Medicine Shanghai 200025 China

**Keywords:** fibrosis, myofibroblast, nanoparticles, profibrotic cytokine

## Abstract

Fibrotic diseases remain a substantial health burden with few therapeutic approaches. A hallmark of fibrosis is the aberrant activation and accumulation of myofibroblasts, which is caused by excessive profibrotic cytokines. Conventional anticytokine therapies fail to undergo clinical trials, as simply blocking a single or several antifibrotic cytokines cannot abrogate the profibrotic microenvironment. Here, biomimetic nanoparticles based on autologous skin fibroblasts are customized as decoys to neutralize multiple fibroblast‐targeted cytokines. By fusing the skin fibroblast membrane onto poly(lactic‐*co*‐glycolic) acid cores, these nanoparticles, termed fibroblast membrane‐camouflaged nanoparticles (FNPs), are shown to effectively scavenge various profibrotic cytokines, including transforming growth factor‐*β*, interleukin (IL)‐11, IL‐13, and IL‐17, thereby modulating the profibrotic microenvironment. FNPs are sequentially prepared into multiple formulations for different administration routines. As a proof‐of‐concept, in three independent animal models with various organ fibrosis (lung fibrosis, liver fibrosis, and heart fibrosis), FNPs effectively reduce the accumulation of myofibroblasts, and the formation of fibrotic tissue, concomitantly restoring organ function and indicating that FNPs are a potential broad‐spectrum therapy for fibrosis management.

## Graphical abstract

Fibroblast‐mimicking nanoparticles are developed using autologous skin fibroblast membrane and poly(lactic‐*co*‐glycolic) acid nanoparticles. These biomimetic nanoparticles are shown to effectively scavenge various profibrotic cytokines and reduce the accumulation of myofibroblasts and the formation of fibrotic tissue, concomitantly restoring organ function and indicating their potential as a broad‐spectrum therapy for fibrosis management.

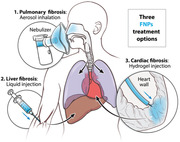

## 1 Introduction

Fibrosis, or disordered fibrotic tissue formation, is characterized by the abnormal fibroblast activation that induces excessive extracellular matrix (ECM) remodeling and primarily accounts for multiple organ dysfunctions.^[^ ^1^ ^]^ The pervasive occurrence of fibrosis in almost all diseases generates a large healthcare burden worldwide. However, the clinical benefits of antifibrotic therapy through small molecules, such as pirfenidone and nintedanib, are usually offset by their modest therapeutic efficacy, limited indications and severe side effects.^[^ ^2^ ^]^ Therefore, alternative clinical intervention modalities to target fibrosis are urgently needed.

Considering the central role of myofibroblast activation and proliferation in fibrosis establishment,^[^ ^3^ ^]^ recent breakthroughs have focused on the ablation of progressive myofibroblast activation through autologous cell‐based therapy. For example, autologous chimeric antigen receptor (CAR) T cell therapy to specifically kill myofibroblasts has achieved unprecedented success in resolving multiorgan fibrosis.^[^ ^4^ ^]^ However, the clinical translation of genetically edited cell therapies may be limited by the exorbitant cost and concomitant immunotoxicity.^[^ ^5^ ^]^ Therefore, further efforts to develop an alternative autologous cell‐based therapeutic modality with low cost and satisfactory biocompatibility are also needed.

Instead of directly killing myofibroblasts, specific blockade of myofibroblast activation represents a promising alternative strategy. Notably, cytokines like transforming growth factor‐*β* (TGF‐*β*) family proteins, interleukin (IL)‐11, IL‐13, and IL‐17 have been shown to exert critical roles in mediating fibrosis.^[^ ^6^ ^]^ Although some of the anticytokine therapies have been approved by the FDA with promising results (such as tocilizumab), which brings a silver lining to the refractory medical issues, Nonetheless, some of them still suffered from unsatisfied clinical outcomes.^[^ ^7^ ^]^ This failure is mainly because: 1) fibrotic disorders involve multiple cytokines, and simple inhibition of a single or a few types of cytokines may not be sufficient; and 2) off‐target inhibition of these cytokines may induce severe side effects. Therefore, next‐generation therapies are expected to use a broad‐spectrum and locally applied anticytokine strategy to target the overall fibrotic microenvironment.

**Figure 1:**
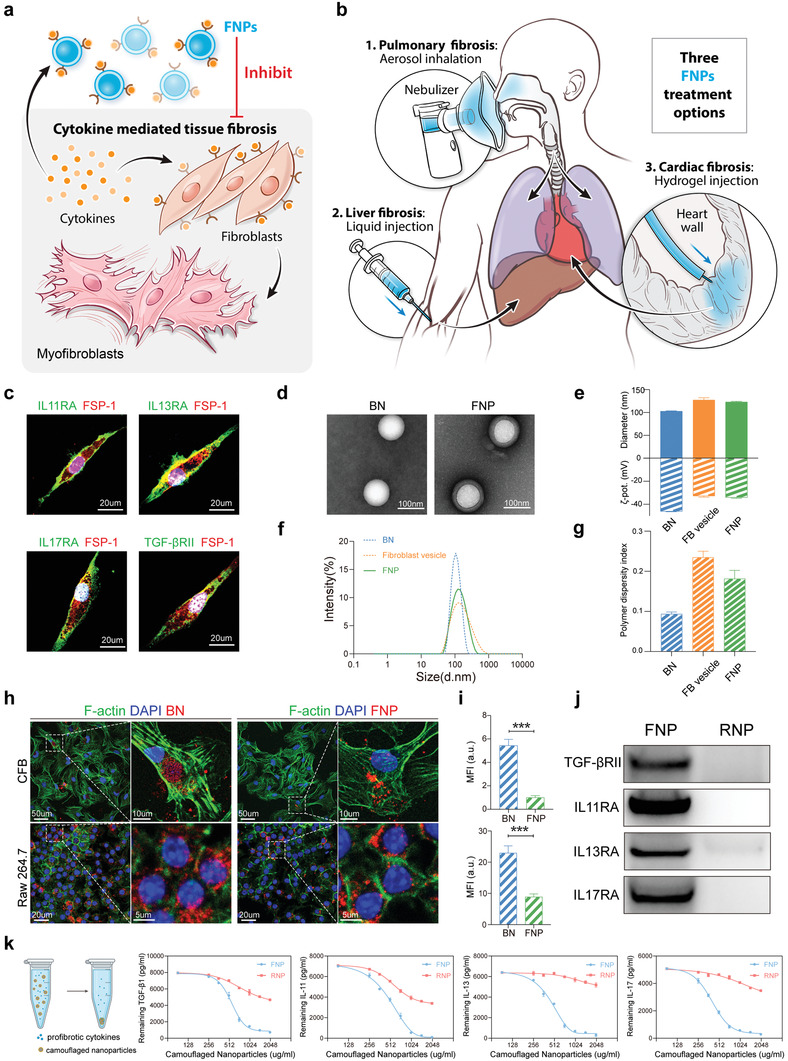
Fabrication and characterization of FNPs. a,b) Schematic of FNPs for treating fibrosis. (a) FNPs function as decoys to capture various cytokines and inhibit differentiation of fibroblasts to myofibroblasts. b) FNPs are prepared into multiple formulations, including aerosol, liquid, and hydrogel to treat lung, liver, and cardiac fibrosis. c) Representative confocal images of skin fibroblasts labeled with fibroblast specific protein‐1 (FSP‐1), IL11RA, IL13RA, IL17RA, and TGF*β*RII. Nucleus was labeled with DAPI (4′,6‐diamidino‐2‐phenylindole). d) TEM images of bare nanoparticles (BNs) and FNPs negatively stained with uranyl acetate. e) Hydrodynamic size (diameter, nm) and zeta potential (*ζ*‐pot, mV). f) Size distribution curves and g) PDI of bare nanoparticles, fibroblast vesicles, and FNPs (*n* = 3 biologically independent samples). h) Representative confocal images showing internalization of bare nanoparticles (red) and FNPs (red) by mouse primary CFB (labeled with phalloidin, green) and Raw 264.7 cells (labeled with phalloidin, green). i) Mean fluorescence intensity (MFI) of bare nanoparticles and FNPs internalized by mouse primary CFBs (top) and Raw 264.7 cells (bottom) (*n* = 3 biologically independent samples). j) Western blot of TGF‐*β*IIR, IL11RA, IL13RA, and IL17RA in FNPs and RNPs. k) Cytokine binding capacity of FNPs and RNPs with TGF‐*β*1, IL11, IL13, and IL17 (*n* = 3 biologically independent samples). The data are expressed as mean ± s.d. (i) Data were analyzed by two‐tailed Student's *t*‐test, ***p < 0.001.

Here, we developed autologous skin fibroblast‐based therapy to effectively attenuate multiorgan fibrosis. Inactivated autologous skin fibroblasts with intact membrane receptors are prepared in a facile, robust, and economically feasible manner. Endogenous receptors function as decoys to regulate the action of cytokines, as they can recognize, sequester, and scavenge certain cytokines but are incapable of triggering signal transduction (**Figure** **1**a). The membrane decoy is supported by a poly(lactic‐*co*‐glycolic) acid (PLGA)‐based nanoparticle cores, termed fibroblast membrane‐camouflaged nanoparticles (FNPs), to enhance stability and facilitate administration. We then examined the competitive binding of multiple profibrotic cytokines with FNPs in vitro, and the antifibrotic efficacy of FNPs in vivo was confirmed through three independent animal models with various organ fibrosis (liver fibrosis, lung fibrosis, and heart fibrosis), which demonstrates its promising clinical potential (Figure 1b).

## 2 Results

### 2.1 Fabrication and Characterization of FNPs

A schematic representation of the fabrication of FNPs is shown in Figure S1 of the Supporting Information. In brief, mouse skin fibroblasts were first isolated from the tail tip and expanded in vitro. Immunofluorescence imaging confirmed the expression of various cytokine receptors, including IL11RA, IL13RA, IL17RA, and TGF‐*β*RII, on skin fibroblasts (Figure 1c). Skin fibroblasts were then harvested, homogenized, and subjected to repeated centrifugations to obtain purified membranes. The membranes were coated onto PLGA cores through a sonication process to form FNPs. When visualized with transmission electron microscopy (TEM), FNPs showed a spherical core–shell structure that indicated unilamellar membrane coatings over the polymeric cores (Figure 1d). Dynamic light scattering (DLS) revealed that FNPs were ≈20 nm larger than the uncoated PLGA nanoparticles (Figure 1e,f), which is similar to the TEM observations. Moreover, zeta‐potential measurements showed that FNPs possessed a similar surface charge to that of fibroblast vesicles (Figure 1e). FNPs possessed a polymer dispersity index (PDI) of 0.18 (Figure 1g), indicating a homogenous population of nanoparticles, and suggesting acceptability for clinical use.^[^ ^8^ ^]^ To optimize the membrane coating efficiency, FNPs were prepared with different membrane protein‐to‐polymer weight ratios as previously described.^[^ ^9^ ^]^ After adjusting with a 1× PBS solution, no apparent size increase was observed in FNPs prepared with a membrane protein‐to‐polymer weight ratio greater than 1:1 (Figure S2, Supporting Information), and this formulation was used for subsequent studies. After their synthesis, FNPs were stored at 4 °C and demonstrated superior stability within 7 days, as monitored by DLS (Figure S3, Supporting Information). Moreover, to assure the physicochemical and biological repeatability of FNPs, a set of quality assurance standards for their manufacturing was developed as previously described^[^ ^10^ ^]^ (Table S1, Supporting Information).

Subsequently, we examined the internalization of DiD‐labeled FNPs and DiD‐labeled PLGA nanoparticles by primary cardiac fibroblasts (CFBs) and macrophages (RAW 264.7 cells). FNPs showed significantly decreased uptake by both cell lines compared to the bare PLGA nanoparticles (Figure 1h,i). However, macrophages showed a higher internalization efficiency of FNPs than CFBs, indicating the potential clearance of FNPs by macrophages in vivo. To evaluate the safety of FNPs, PBS or FNPs (20 mg kg^−1^) were intravenously injected into healthy mice. After 24 h, compared to mice receiving PBS, mice receiving FNPs showed no statistically significant differences in immune cell count (including neutrophils, lymphocytes, and monocytes) or the levels of proinflammatory cytokines (including IL‐6 and TNF‐*α*), indicating that FNPs did not provoke immune responses in vivo (Figure S4, Supporting Information). Next, western blotting showed that the FNPs contained various receptors responsible for cytokine binding, including TGF‐*β*RII, IL11RA, IL13RA, and IL17RA (Figure 1j). As a control, we further prepared red blood cell membrane‐camouflaged nanoparticles (RNPs) with a spherical core–shell structure, size distribution, and PDI similar to those of FNPs (Figure S5, Supporting Information). However, western blotting showed that RNPs had low‐to‐no expression of the aforementioned cytokine receptors (Figure 1j). We then tested the binding capacity of FNPs to various profibrotic cytokines, including IL11, IL13, IL17A, and TGF‐*β*1, which play prominent roles in fibrosis progression.^[^ ^1a^ ^]^ We found that FNPs but not RNPs, effectively neutralized all four cytokines in a dose‐dependent manner (Figure 1k). Taken altogether, our findings demonstrate the successful fabrication of FNPs and their cytokine neutralization ability in vitro.

### 2.2 FNPs Suppress TGF‐*β*1‐Induced Myofibroblast Differentiation

In the profibrotic environment, several progenitor cell types, such as resident fibroblasts, epithelial cells, and endothelial cells, can be activated and differentiate into myofibroblasts.^[^ ^1c^ ^]^ To examine whether FNPs could suppress the profibrotic effect of TGF‐*β*1, different resident mesenchymal cells from different organs, including lung fibroblasts, CFBs, and hepatic stellate cells, were used to establish in vitro fibrosis models.

**Figure 2:**
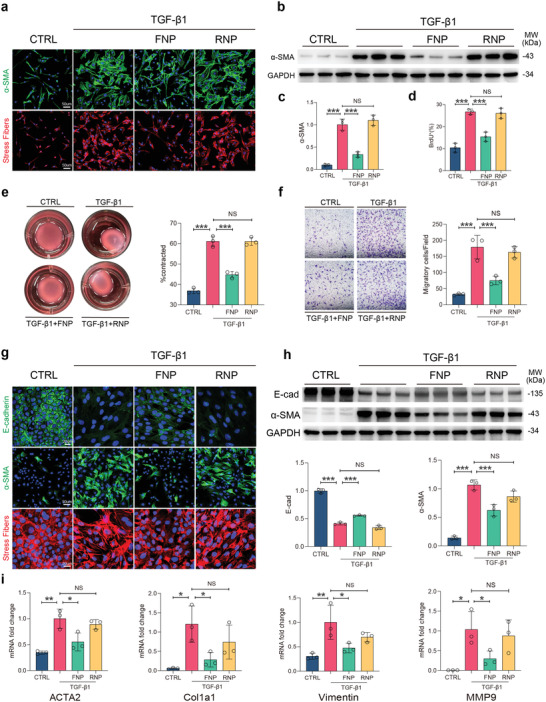
FNPs attenuate TGF‐*β*1‐induced myofibroblast differentiation. a–d,f) Mouse primary lung fibroblasts were incubated with TGF‐*β*1 (5 ng mL^−1^), FNPs, RNPs or without stimulation (CTRL) for 24 h (*n* = 3 biologically independent samples). (a) Representative immunofluorescent images showing *α*‐SMA and stress fibers, nuclei were labeled with DAPI. (b,c) Western blot images and quantification of *α*‐SMA expression (normalized to GAPDH). (d) Percentage of BrdU positive cells using BrdU incorporation assays. e) Mouse primary lung fibroblasts were seeded in collagen gel and incubated with TGF‐*β*1 (5 ng mL^−1^), FNPs, RNPs or without stimulation (CTRL) for 48 h and the area of contraction was quantified (*n* = 3 biologically independent samples). (f) Representative migratory images and quantification of mouse primary lung fibroblasts by Transwell assay after 24 h of migration. g–i) NMuMG cells were incubated with TGF‐*β*1 (5 ng mL^−1^), FNPs, RNPs or without stimulation (CTRL) for 24 h (*n* = 3 biologically independent samples). (g) Representative immunofluorescent images showing E‐cadherin, *α*‐SMA, and stress fibers, nucleus were labeled with DAPI. (h) Western blot images and quantification (normalized to GAPDH) of E‐cadherin and *α*‐SMA expression. (i) Relative mRNA expressions (normalized to GAPDH) of ACTA2, Col1a1, vimentin, and MMP9. The data are expressed as mean ± s.d. Data were analyzed by one‐way ANOVA with Tukey's post hoc test, NS indicates not significant, *p < 0.05, **p < 0.01, ***p < 0.001.

Stimulation of resting lung fibroblasts with TGF‐*β*1 promoted the expression of *α*‐smooth muscle actin (*α*‐SMA) and the formation of stress fibers (**Figure** **2**a), indicating myofibroblasts differentiation. Treatment with FNPs significantly ameliorated the stimulatory effects of TGF‐*β*1‐induced fibroblast activation, which manifested as decreased *α*‐SMA expression and stress fiber formation. By contrast, RNPs did not show cytokine neutralizing effects (Figure 2a). This result suggests that FNPs recognized and competitively bound TGF‐*β*1 through membrane receptors, while RNPs, which lack TGF‐*β*1 receptors, showed inadequate binding capacity. Consistent with the immunofluorescence results, western blot analysis also confirmed that FNPs but not RNPs could inhibit TGF‐*β*1‐induced *α*‐SMA expression (Figure 2b,c). A Similar phenomenon was also observed in CFBs (Figure S6, Supporting Information) and hepatic stellate cells (Figure S7, Supporting Information), suggesting potential broad‐spectrum antifibrotic effects across different organs. Activated fibroblasts are characterized as proliferative, contractive, and migrative/invasive.^[^ ^3^ ^]^ Through bromodeoxyuridine (BrdU) incorporation assays, collagen contractions assays, and transwell migration assays, we found that TGF‐*β*1 could enhance the proliferative capacity (Figure 2d), contractive capacity (Figure 2e), and migratory capacity (Figure 2f) of lung fibroblasts. In the presence of FNPs, the aforementioned effects induced by TGF*β*‐1 were significantly inhibited, and this effect was marginally robust in RNPs (Figure 2d–f).

In addition to resident mesenchymal cells, myofibroblasts can also be derived from epithelial cells through epithelial to mesenchymal transition (EMT). To explore whether FNPs affects this process, we used a canonical TGF‐*β*1‐induced EMT assay using the mouse mammary gland (NMuMG) epithelial cell line.^[^ ^3^, ^11^ ^]^ Upon stimulation with TGF‐*β*1, adherence junctions between NMuMG cells were disrupted, which manifested as downregulated expression of E‐cadherin. The expression of *α*‐SMA increased and F‐actin was rearranged from cortical to a stress fiber distribution (Figure 2g,h). TGF‐*β*1 also enhanced the mRNA expression of ACTA2, vimentin, Col1a1, and MMP9 (Figure 2i), indicating that NMuMG cells had transitioned from an epithelial to a mesenchymal state, which is myofibroblast transdifferentiation. The addition of FNPs to the culture medium attenuated all these effects, whereas this outcome was not observed with RNPs (Figure 2g–i). Finally, we confirmed that FNPs inhibits TGF‐*β*1‐induced endothelial to mesenchymal transition (EndoMT) using mouse aortic endothelial cells (Figure S8, Supporting Information). Taken altogether, these results demonstrated that FNPs attenuated TGF‐*β*1‐induced myofibroblast differentiation in multiple progenitor cells.

### 2.3 Intratracheal Administration of FNPs Ameliorated Bleomycin‐Induced Lung Fibrosis

A bleomycin‐induced murine lung fibrosis model was used to test the therapeutic potential of FNPs.^[^ ^12^ ^]^ Fluorescently labeled FNPs or RNPs were administered intratracheally to mice through a commercial microsprayer to evaluate their corresponding biodistribution. As shown in Figure S9 of the Supporting Information, FNPs were uniformly distributed in the pulmonary mesenchyme without obvious retention in the bronchia. A single dose of FNPs (50 µL at 2 mg mL^−1^) showed durable retention in the lungs for 1 week (Figure S10a,b, Supporting Information). FNPs were also detected in the liver, spleen, and kidney (Figure S10c,d, Supporting Information), indicating their clearance by the reticuloendothelial system and transrenal metabolism. The clearance of FNPs by macrophages was also supported by the fact that fluorescently labeled FNPs colocalized with F4/80^+^ cells in bleomycin‐treated lungs (Figure S11, Supporting Information). These results suggest the pharmaceutical potency of FNPs for lung fibrosis in vivo.

**Figure 3:**
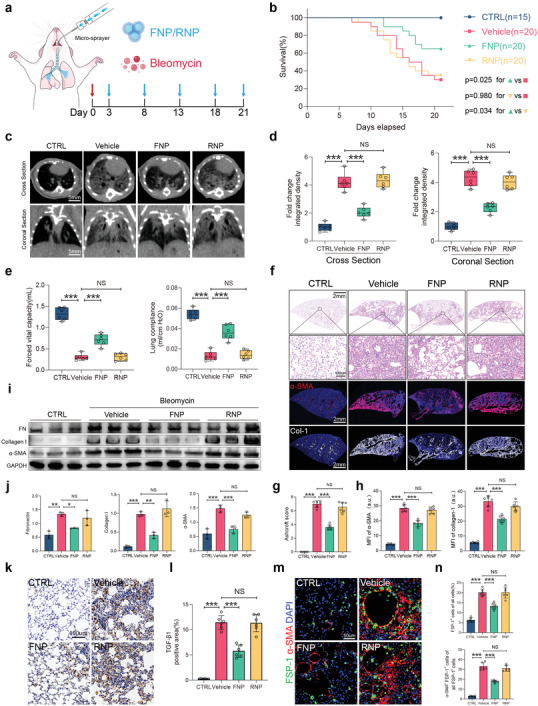
Intratracheal administration of FNPs attenuates bleomycin‐induced lung fibrosis. a) Experimental scheme of bleomycin‐treated mice administered with FNPs, RNPs (50 µL at 2 mg mL^−1^) or vehicle. b) Percent survival during 21 days of treatment after bleomycin injury. c) Representative cross‐section and coronal sections of lung micro‐CT images on day 21. d) Quantification of lung fibrosis severity by the integrated intensity of CT images (*n* = 6 biologically independent mice per group). e) Forced vital capacity and lung compliance was measured on day 21. f) Representative H&E staining and immunofluorescence staining of *α*‐SMA, collagen I from different treatment groups. g) Ashcroft scores evaluated from H&E staining (*n* = 6 biologically independent mice per group. h) Quantification of MFI of *α*‐SMA and collagen I (*n* = 6 biologically independent mice per group). i) Western blot analysis and quantification j) of fibronectin, collagen I, and *α*‐SMA expression from bleomycin‐induced fibrotic lungs of different treatment groups (*n* = 3 biologically independent mice per group). k) Representative immunohistochemistry staining of TGF‐*β*1 and l) percentage of TGF‐*β*1 positive area from different treatment groups (*n* = 6 biologically independent mice per group). m) Representative immunofluorescence staining of FSP‐1 (green) and *α*‐SMA (red), nuclei were labeled with DAPI. n) Percentage of cells that was FSP1^+^ (top) and percentage of FSP1+ cells that was *α*‐SMA^+^ (bottom) for each group. (g,h,j,l,n) Data are expressed as mean ± s.d. (d,e) Data are presented as box‐and‐whisker plots. Survival distributions were estimated by the Kaplan–Meier method and compared by the log‐rank test. Data were analyzed by one‐way ANOVA with Tukey's post hoc test, NS indicates not significant, *p < 0.05, **p < 0.01, ***p < 0.001.

Subsequently, we tested the antifibrotic effects of FNPs on lung fibrosis. The mice were intratracheally administered bleomycin to induce lung fibrosis at day 0. From day 3, which was considered the early fibrogenic phase,^[^ ^6e^ ^]^ the mice underwent intratracheal inhalation of FNPs (50 µL at 2 mg mL^−1^), RNPs or vehicle (PBS solution) via a microsprayer every 5 days until the end of observation (**Figure** **3**a). FNP treatment significantly reduced the TGF‐*β*1 levels in bronchoalveolar lavage fluid compared to vehicle or RNPs on day 4 (one day after the first treatment) (Figure S12, Supporting Information). Kaplan–Meier curves showed that FNP treatment markedly prolonged the overall survival rates compared to the other treatments (FNP: 65%, vehicle: 30%, RNP: 35%) (Figure 3b). Micro‐CT scanning indicated that bleomycin exposure caused robust injury and fibrosis in the vehicle groups by day 21, which were significantly diminished in FNP‐treated mice (Figure 3c,d). FNP‐treated mice also exhibited preserved lung functions, as monitored by forced vital capacity, lung compliance (Figure 3e), forced expiratory volume, expiratory reserve volume, peak expiratory flow, and total lung resistance (Figure S13, Supporting Information).

Histological analysis showed that 21 days after bleomycin injury, the lungs of mice in the vehicle group exhibited severe distortion of alveolar structure and the formation of honeycomb‐like fibrous masses (Figure 3f). FNP treatment effectively reduced the fibrotic area and preserved the normal alveolar structure (Figure 3f), as demonstrated by the reduced Ashcroft scores (Figure 3g). Bleomycin also increased *α*‐SMA and collagen I staining in the lungs, indicating the accumulation of myofibroblasts and deposition of ECM, which were significantly reduced by FNP treatment but not RNP treatment (Figure 3f,h). In line with these observations, western blot analysis confirmed a marked reduction in fibronectin, collagen I and *α*‐SMA in the lungs from FNP‐treated mice (Figure 3i,j). Likewise, a reduction in the total lung hydroxyproline level was also observed (Figure S14, Supporting Information). Furthermore, in FNP‐treated mice, the level of the critical profibrotic cytokine TGF‐*β*1 (demonstrated by the TGF‐*β*1‐positive area) was significantly reduced (Figure 3k,l). Analysis of FSP‐1/*α*‐SMA double labeling revealed that FNP administration reduced the percentage of FSP‐1‐positive cells, and the percentage of FSP‐1/*α*‐SMA double‐positive cells (Figure 3m,n), possibly reflecting the reduction in the proliferation of lung fibroblasts and their differentiation into myofibroblasts.

### 2.4 Intravenous Administration of FNPs Ameliorated Carbon Tetrachloride (CCl_4_)‐Induced Liver Fibrosis

**Figure 4:**
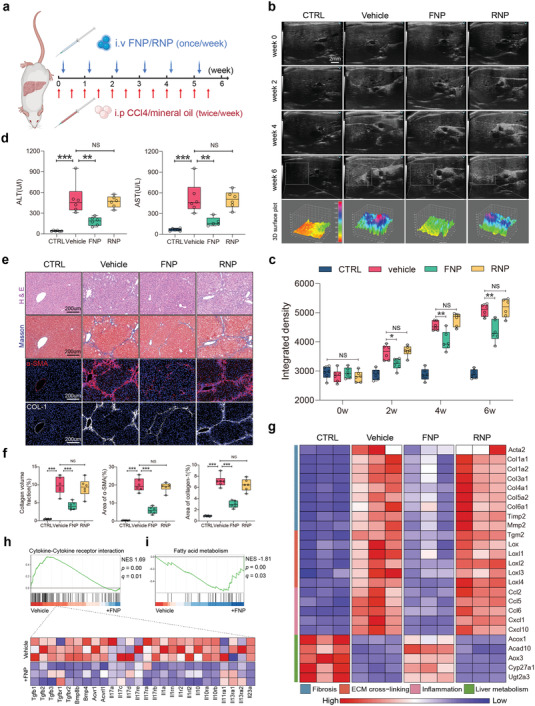
Intravenous administration of FNPs attenuates CCl_4_‐induced liver fibrosis. a) Schematic representation of CCl_4_‐induced liver fibrosis and treatment timelines for FNPs, RNPs (100 µL at 2 mg mL^−1^) or vehicle. b) Representative ultrasound images of mice livers from different treatment groups before and 2, 4, and 6 weeks after the initiation of CCl_4_ injections. The 3D surface plots within the gray squares correspond to the echogenic intensity and uniformity in the liver. c) Quantification of liver fibrosis severity by integrated intensity of ultrasound images (*n* = 6 biologically independent mice per groups). d) Serum liver function tests of mice from different treatment groups (*n* = 6 biologically independent mice per groups). e) Representative H&E staining, Masson's trichrome staining, and immunofluorescence staining of *α*‐SMA, collagen I from different treatment groups. f) Quantification of collagen volume fraction from Masson's trichrome staining and area% of *α*‐SMA and collagen I (*n* = 6 biologically independent mice per groups). g) Heat map of profibrotic, proinflammatory, ECM cross‐linking‐related and liver metabolism‐related gene expressions. h,i) GSEA for indicated MSigDB‐defined gene clusters. Data are presented as box‐and‐whisker plots. Data were analyzed by one‐way ANOVA with Tukey's post hoc test, NS indicates not significant, *p < 0.05, **p < 0.01, ***p < 0.001.

We further extended the application of FNPs to liver fibrosis. The liver sequesters a majority of the nanomaterials administered to the body due to its unique organ microstructure and blood flow dynamics,^[^ ^13^ ^]^ which enables FNPs with natural liver‐targeting ability. As shown in Figure S15a,b of the Supporting Information, we confirmed that most FNPs and RNPs accumulated in the liver after intravenous administration in mice. Continuous in vivo fluorescence observation revealed that FNPs or RNPs persisted in the liver for more than 1 week (Figure S15c,d, Supporting Information). We subsequently evaluated the antifibrotic efficacy of FNPs. Mice were subjected to 6 weeks of CCl_4_ to establish liver fibrosis and received treatment with FNPs, RNPs or vehicle every 7 days (**Figure** **4**a). We monitored the liver by ultrasonography at various time points (weeks 0, 2, 4, 6) throughout the study. The intensity and heterogeneity of liver echogenicity in the vehicle group gradually increased with fibrosis development, and large areas of fibrous bands were observed adjacent to the portal vein area (Figure 4b). Weekly intravenous injection of FNPs effectively slowed the progression of liver fibrosis, which was characterized by reduced and more homogeneous liver echogenicity (Figure 4b,c). Serum concentrations of alanine transaminase and aspartate transaminase, which are indicators of liver injury, were also decreased in the FNP treatment group (Figure 4d). Consistent with the ultrasonic manifestations, histological analysis confirmed that the administration of FNPs produced a marked reduction in fibrotic areas and downregulated *α*‐SMA expression, collagen I deposition (Figure 4e,f) and total liver collagen content (Figure S16, Supporting Information) compared to treatment with vehicle or RNPs. Furthermore, we performed RNA sequencing to profile the therapeutic effects of FNPs. FNPs reduced the expression of profibrotic, proinflammatory, and ECM cross‐linking‐related genes and restored liver metabolism gene expression (Figure 4g). Subsequent gene set enrichment analysis (GSEA) revealed that FNPs inhibited the cytokine–cytokine receptor interaction, chemokine production, and inflammatory response‐related gene signature (Figure 4h; Figure S17, Supporting Information) and restored the liver metabolism (including fatty acid, bile acid, glycogen, tyrosine, and drug metabolism)‐related molecular signatures database (MSigDB)‐defined gene set (Figure 4i; Figure S17, Supporting Information).

### 2.5 Intramyocardial Injection of FNP‐Alginate Hydrogel (AH) Attenuated Regional Fibroblast Activation and Collagen Deposition and Altered Collagen Subtype

We then tested the potential use of FNPs in attenuating cardiac fibrosis. In response to ischemic cardiac injury, CFBs rapidly proliferate and undergo myofibroblast differentiation, resulting in increased ECM deposition and the formation of collagen‐based scars to maintain the structural integrity of infarcted myocardium.^[^ ^14^ ^]^ This fibrotic response may result in cardiac stiffening and functional deterioration, and is the major pathogenesis of heart failure.^[^ ^15^ ^]^ We hypothesized that FNPs also exert antifibrotic effects on the infarcted myocardium and improve the overall cardiac function after myocardial infarction (MI). We used local intramyocardial delivery of FNPs to the fibrotic myocardium via an injectable AH, which is widely used in clinical trials.^[^ ^16^ ^]^

**Figure 5:**
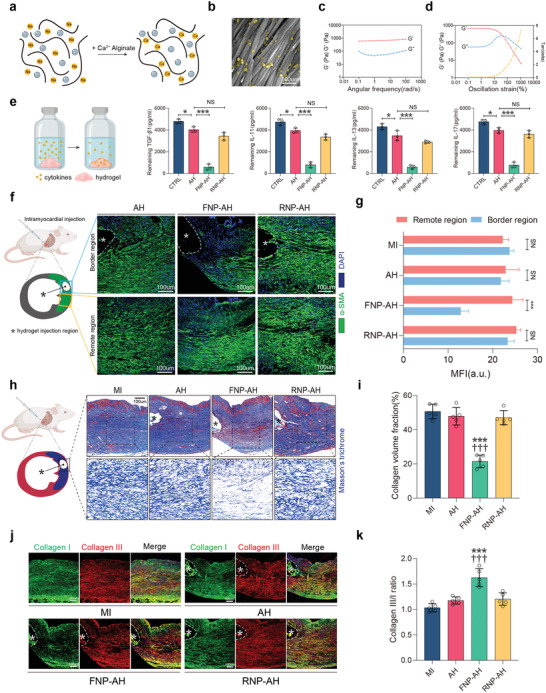
Intramyocardial injection of FNP‐AH attenuates regional fibroblast activation and collagen deposition and alters collagen subtype. a) Schematic representation of the forming process of FNP‐AH composite. b) A pseudocolored SEM image of the FNP‐AH (FNPs colored in yellow). c) Oscillatory frequency sweeps of FNP‐AH. d) Oscillatory strain sweeps of FNP‐AH. e) Cytokine binding capacity of AH, FNP‐AH, RNP‐AH with TGF‐*β*1, IL11, IL13, and IL17 (*n* = 3). f) Representative immunofluorescent staining of *α*‐SMA and g) quantification of mean fluorescent intensity in border region and remote region relative to the hydrogel injection site on day 7 (*n* = 5 biologically independent mice per groups). h) Representative Masson's trichrome staining and i) quantification of collagen volume fraction on day 7 (*n* = 5 biologically independent mice per groups). j) Representative immunofluorescent staining for collagen I (green), collagen III (red), and DAPI on day 7 and k) the ratio of collagen III to collagen I (*n* = 5 biologically independent mice per groups). (f,h,j) Asterisk (*) indicates the hydrogel injection region. The data are expressed as mean ± s.d. g) Data were analyzed by two‐tailed Student's *t*‐test, NS indicates not significant, ***p < 0.001. i,k) Data were analyzed by one‐way ANOVA with Tukey's post hoc test, ***p < 0.001 compare to the MI group, ^†††^ *p* < 0.001 compare to the AH group.

As shown in **Figure** **5**a, FNPs were first embedded in sodium alginate solution, which then interacted with a calcium alginate suspension to form ionic bridges between alginate chains, leading to the formation of FNP‐alginate hydrogel composites. Scanning electron microscopy (SEM) indicated the even distribution of FNPs in the alginate scaffold (Figure 5b). Frequency sweep measurements showed the FNP‐AH possessed constant elastic modulus (*G* ^′^) values ≈10 times higher than the viscous modulus (*G* ^″^) values, indicating the formation of a stable hydrogel (Figure 5c). Furthermore, FNP‐AH exhibited shear‐thinning behavior in the strain‐dependent oscillatory rheology tests, as demonstrated by the rapid decrease in *G*′ values with increasing strain (Figure 5d).

Next, we incubated the AH, FNP‐AH, and RNP‐AH with various profibrotic cytokine solutions to assess the binding capacity of these hydrogels in a biologically relevant fibrotic context. The results demonstrated the high binding capacity of FNP‐AH but insufficient binding capacities of AH and RNP‐AH to TGF‐*β*1, IL11, IL13, and IL17 (Figure 5e). To further test the antifibrotic effects of FNP‐AH through its cytokine scavenging capacity, we used a mouse model of MI. Single‐point injection of hydrogels in the middle of the infarct area was performed after ligation of the left anterior descending (LAD) coronary artery. Seven days after the initiation of ischemic injury, when acute inflammation had already subsided and fibrosis gradually formed, we performed histological analysis to observe fibroblast activation, collagen deposition, and collagen subtypes in the region adjacent to the injection site. As shown in Figure 5f,g, in response to the acute inflammatory process, fibroblasts proliferated and became activated, leading to the accumulation of numerous *α*‐SMA‐positive myofibroblasts in the infarct region. However, the number of *α*‐SMA positive cells was significantly reduced in the region adjacent to the FNP‐AH injection site (Figure 5f,g), suggesting that FNP‐AH scavenged ambient profibrotic cytokines and prevented neighboring fibroblasts from activating and differentiating. Correspondingly, collagen deposition, which is mainly caused by myofibroblasts, was also markedly reduced in the area adjacent to the FNP‐AH injection site (Figure 5h,i). Furthermore, a higher percentage of elastic collagen III fibers than rigid collagen I fibers was observed in the FNP‐AH groups, indicating that FNP‐AH improves scar compliance (Figure 5j,k).

### 2.6 FNP‐AH Composites Improved Cardiac Function, Reduced Fibrotic Area and Limited Adverse Cardiac Remodeling after MI

**Figure 6:**
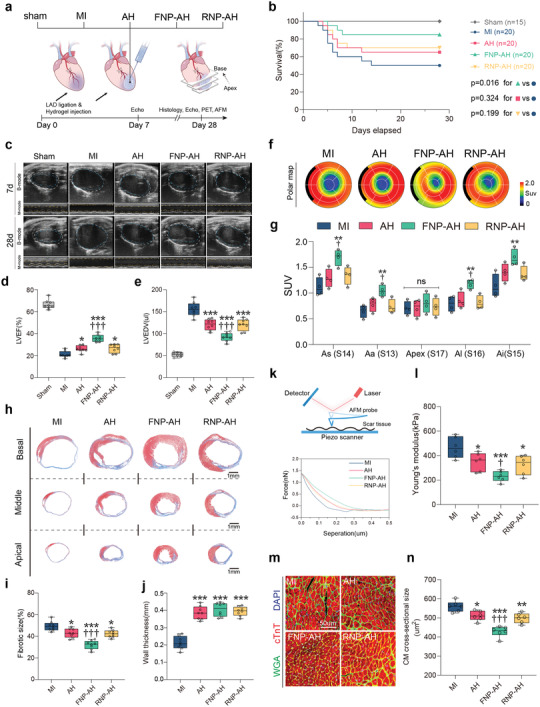
FNP‐AH improves cardiac function, reduces fibrotic area, and limits adverse cardiac remodeling after MI. a) Schematic representation of the study design. b) Percent survival during 28 days after MI. c) Representative echocardiography images on day 7 and day 28. d) LVEF and e) LVEDV assessed by echocardiography on day 28 (*n* = 8 biologically independent mice per group). f) Representative 17‐segment polar map via ^18^F‐FDG PET imaging on day 28. g) Quantification of SUV of ^18^F‐FDG via PET imaging (*n* = 4 biologically independent mice per group). As, apical septal; Aa, apical anterior; Al, apical lateral; Ai, apical inferior. h) Representative Masson's trichrome staining on day 28. i) Quantification of fibrotic size and j) wall thickness based on the Masson's trichrome staining (*n* = 8 biologically independent mice per group). k–l) Measurement of the Young's modulus of scar tissue by AFM indentation. (k) Schematic representation of AFM indentation (top) and representative force curve graphic (bottom). (l) Quantification of Young's modulus for each group (*n* = 6 biologically independent mice per group). m) Representative immunostaining of wheat germ agglutinin (WGA), cardiac troponin T (cTnT), and DAPI to visualize the cross‐section of cardiomyocytes in the infract border zone. n) Quantification of cross‐sectional area of cardiomyocytes based on the WGA staining (*n* = 6 biologically independent mice per group). Data are presented as box‐and‐whisker plots. Survival distributions were estimated by the Kaplan–Meier method and compared by the log‐rank test. Data were analyzed by one way ANOVA with Tukey's post hoc test, *p < 0.05, **p < 0.01, ***p < 0.001 compare to the MI group, ††*p* < 0.01, †††*p* < 0.001 compare to the AH group.

Next, we sought to examine its therapeutic effects on overall cardiac function and fibrotic response. We conducted the study on a longer time scale (28 days). MI was first induced by the ligation of LAD coronary artery, followed by multipoint injections of FNP‐AH, RNP‐AH or AH in the ischemic area and border area (**Figure** **6**a). FNPs showed durable retention in the myocardium even after 2 weeks (Figure S18, Supporting Information). Kaplan–Meier analysis demonstrated the application of FNP‐AH but not AH or RNP‐AH improved survival in the MI group (Figure 6b). On day 7, proteomic analysis demonstrated that FNP‐AH treatment reduced cytokine levels and cardiac fibroblast activation, thereby reducing ECM cross‐linking and deposition compared to those in the MI group (Figure S19, Supporting Information). We observed a significant improvement in cardiac function, as assessed by left ventricular ejection fraction (LVEF) and left ventricular fractional shortening (LVFS) in the FNP‐AH group and moderate improvements in the AH and RNP‐AH groups compared to the MI group 1 week after injury (Figure S20a, Supporting Information), highlighting the amelioration of ventricular impairment after MI. This effect was maintained for the 4‐week period after MI (Figure 6d; Figure S20b, Supporting Information). Left ventricular dilation, as determined by the left ventricular end‐diastolic diameter (LVEDD) and left ventricular end‐diastolic volume (LVEDV), was also significantly prevented in the FNP‐AH group and moderately prevented in the AH and RNP‐AH groups (Figure 6e; Figure S20c, Supporting Information). The protective effects of bare AH and RNP‐AH were attributed to mechanical and physical support to the damaged cardiac tissue after MI.^[^ ^17^ ^]^ Next, as assessed by positron emission tomography (PET), the FNP‐AH group showed a notably smaller infarct area than the other groups (Figure S21, Supporting Information), and cardiomyocyte viability in the infarct border zone, as determined by the ^18^F‐FDG standardized uptake value (SUV), was significantly increased in the FNP‐AH group (Figure 6f,g).

Next, we performed histological analysis to evaluate the effects of FNP‐AH on ventricular fibrosis and remodeling. Masson's trichrome staining demonstrated that fibrosis was significantly ameliorated in the FNP‐AH group and also moderately ameliorated in the AH and RNP‐AH groups (Figure 6h,i). Wall thickness was significantly increased in the FNP‐AH, RNP‐AH, and AH groups compared to that in the MI group (Figure 6j). Furthermore, collagen deposition and fiber morphology were directly visualized using a multiphoton laser scanning microscope that captures the second harmonic generation (SHG) signals excited from collagen fibrils. Collagen fibrils in the fibrosis area of the MI group had a highly directed and isotropic architecture, as shown by the SHG images. By contrast, the FNP‐AH group exhibited a sparser and anisotropic collagen fiber arrangement (Figure S22a, Supporting Information). Quantitation of fibril features in SHG images revealed enhanced skewness and kurtosis in FNP‐AH compared to that in the other groups, indicating thinner and dispersed collagen fibers, which may lead to reduced tissue stiffness (Figure S22b, Supporting Information). Thus, we also evaluated the stiffness of fibrotic tissue using atomic force microscopy (AFM), which showed a significant decrease in the Young's modulus in FNP‐AH group and a moderate decrease in the RNP‐AH and AH groups compared to the MI group (Figure 6k,l). Because increased tissue stiffness and cardiac dysfunction can lead to a hypertrophic response in cardiomyocytes, we also observed that cardiomyocytes located in the border zone of the FNP‐AH group had a considerably lower cross‐sectional area than the other groups (Figure 6m,n).

## 3 Discussion

Fibrotic disorders affect virtually every organ system and contribute to an estimated 45% of all deaths in the industrialized world.^[^ ^1^, ^6^ ^]^ The healthcare burden is further aggravated by the scarcity of therapeutic choices and their limited clinical indications. For example, pirfenidone, one of the few clinically approved drugs for treating idiopathic pulmonary fibrosis, was shown to have notable toxicities.^[^ ^2^ ^]^ Moreover, drug development has been restricted to lung‐related fibrotic diseases.^[^ ^1a^ ^]^ Therefore, further endeavors were made to develop a broad‐spectrum antifibrotic therapy in a simple, facile, and reproducible manner.

A large body of evidence has placed the profibrotic cytokines family in a central position in the pathogenesis of fibrosis generation and progression. Therefore, several antibodies were designed to neutralize profibrotic cytokines. For example, fresolimumab, a pan‐specific TGF‐*β* (including TGF‐*β*1, 2, 3)‐neutralizing antibody, reversed markers of skin fibrosis and improved clinical symptoms in systemic sclerosis patients.^[^ ^18^ ^]^ However, indicated by the failure of recent clinical trials targeting single cytokines or their receptors like IL‐13,^[^ ^7a,b^ ^]^ IL‐1,^[^ ^7c^ ^]^ IL‐6R,^[^ ^7d^ ^]^ specific antibody intervention alone had only a modest effect on reversing fibrosis, highlighting that fibrotic disorders are orchestrated by a variety of cytokines. For example, TGF‐*β* family proteins are regarded as the predominant drivers of fibrosis. TGF‐*β* directly acts on resident fibroblasts to induce activation, proliferation, migration, and the production of ECM components, including collagens and fibronectin.^[^ ^6b^ ^]^ However, IL‐17A increases the expression of TGF‐*β*RII on fibroblasts and enhances neutrophil recruitment and the production of proinflammatory cytokines, including TGF‐*β*, which in turn induces its own expression, indicating a feed‐forward mechanism among cytokines.^[^ ^19^ ^]^

Recently, to address the multiplicity of cytokine targets in diseases, novel broad‐spectrum cytokine neutralization strategies based on various biomaterials (e.g., hydrogels and nanoparticles) have emerged as promising therapeutic approaches to modulate the overall microenvironment in the lesion area. For example, glycosaminoglycan‐based hydrogels were able to scavenge multiple chemokines and cytokines and thus attenuate inflammation and promote healing in chronic wounds.^[^ ^20^ ^]^ Biomimetic nanoparticles that resembled native macrophages and neutrophils were shown to be effective in treating acute sepsis and chronic rheumatic arthritis due to their anticytokine abilities.^[^ ^10^, ^21^ ^]^ In addition, porous silicon nanoparticles were also found to attenuate inflammatory responses and hepatocellular damage after acute liver injury through a similar mechanism.^[^ ^22^ ^]^ The majority of these studies focused on inflammation attenuation, and to the best of our knowledge, no corresponding studies have been conducted to adopt nanoparticles as nanoscavengers for treating fibrosis.

In this study, we developed an antifibrosis therapy that targets multiple cytokines based on autologous skin fibroblasts. Autologous skin fibroblasts can be reasonably obtained from skin grafting, which is a regular clinical protocol for treating patients with burns and ulcers.^[^ ^23^ ^]^ In addition, the satisfaction of biosafety and immunogenicity, which is the preliminary considerations during materials design, can be guaranteed as a result of the host‐derived nature of the major components of the purposed nanosystem. To further facilitate clinical translation, the skin fibroblast membrane was fused onto a nanosized polymeric core composed of the FDA‐approved polymer PLGA. These nanoparticles, termed FNPs, are able to sequester and scavenge various profibrotic cytokines, including TGF‐*β*1, IL11, IL13, and IL17. In vitro, we showed that FNPs can effectively suppress TGF‐*β*1‐induced myofibroblast differentiation from multiple progenitor cells, including fibroblasts, epithelial cells, and endothelial cells. FNPs were able to ameliorate experimentally induced lung, liver, and heart fibrosis, restoring organ functions in different in vivo models. The antifibrotic efficiency of FNPs in vivo may be attributed to scavenging crucial profibrotic cytokines and blocking interactions between multiple cytokines and cytokine receptors. However, it should be noted that those observed antifibrosis effects of FNPs may also be downstream from changes in macrophage phenotype. It has been reported that apoptotic cell‐mimicking nanoparticles promoted an anti‐inflammatory phenotype shift in macrophages and reduced the expression of several important proinflammatory cytokines.^[^ ^24^ ^]^

As undesired biodistribution and off‐target effects may reduce the antifibrotic efficacy of FNPs in vivo, to facilitate potential clinical translation, we used varied routes of administration and formulations to target different organs. We delivered FNPs directly to the mouse lungs via a microsprayer to simulate relevant clinical scenarios in which drugs were inhaled by a nebulizer. Moreover, FNPs can be directly intravenously injected to treat liver fibrosis due to their inherent liver accumulation behavior. Finally, using AH as a carrier, FNPs are directly delivered to the heart via intramyocardial injection. Owing to the robust and stable physiochemical behavior of FNPs, they can be feasibly reformulated based on the clinical requirements, indicating the broad clinical applications of FNPs. More importantly, the results demonstrated that the administration of FNPs at the early fibrogenic phase of different murine disease models improve the overall disease prognosis, even in a long‐term manner, and this may suggest the interruption of initial positive profibrogenic feedback loops could introduce a long‐term fibrotic milieu amelioration, which may hold great clinical value.

There are several limitations to our current study. First, FNPs were administered at the initiation stage of fibrosis, and showing therapeutic efficacy in preventing fibrosis progression, and whether they can reverse fibrosis in an established fibrosis model were not investigated. Second, fibrosis models in our study might not fully recapture the features of clinical patients. Third, despite the promising preclinical therapeutic efficacy of current purposed strategy, the detailed molecular mechanisms are probably multifaceted and still, partly unknown. For example, further studies should be conducted to characterize the changes in macrophage phenotype after administration of FNPs in vivo.

## 4 Conclusion

In conclusion, we developed a facile method for producing antifibrosis agents based on autologous skin fibroblasts. Compared to other autologous cell‐based therapies, the current strategy has distinct advantages in abundant cell resources, relatively low cost and short preparation time (1–2 weeks). However, it should be noted that for patients with a high risk of MI, a cell bank may be established in advance to collect skin fibroblasts to produce adequate fibroblast‐derived membranes for personalized FNP therapy. Furthermore, fibroblasts derived from induced pluripotent stem cells can be used to make FNPs for patients with acute injuries. The proposed strategy may also serve as a conserved mechanism in antifibrosis therapeutic modality design. However, to further facilitate its clinical translation, further efforts should be made to investigate its potential role in reversing fibrosis, expand the neutralizing capacity of the cell membrane via genetic methods and test its therapeutic efficacy in other fibrosis models.

## 5 Experimental Section

All the experimental details are reported in the Supporting Information.

## Conflict of Interest

The authors declare no conflict of interest.

## Supporting information

Supporting Information

Supplemental Movie 1

Supplemental Movie 2

Supplemental Movie 3

## Data Availability

The data that support the findings of this study are available from the corresponding author upon reasonable request.
