# Supplementary material for: Autologous Skin Fibroblast‐Based PLGA Nanoparticles for Treating Multiorgan Fibrosis
Source: Adv Sci (Weinh). 2022 May 23;9(21):2200856. doi: 10.1002/advs.202200856 (PMC9313479; doi:10.1002/advs.202200856)
Supplement: Supplementary file 1 — Supporting Information [file ADVS-9-2200856-s004.pdf]

ADVANCED SCIENCE
Open Access

Supporting Information

for *Adv. Sci.*, DOI 10.1002/advs.202200856

Autologous Skin Fibroblast-Based PLGA Nanoparticles for Treating Multiorgan Fibrosis

Qiang Long, Zehua Liu, Qianwen Shao, Hongpeng Shi, Shixing Huang, Chenyu Jiang, Bei Qian, Yiming Zhong, Xiaojun He, Xiaogang Xiang, Yang Yang, Bing Li, Xiaoxiang Yan, Qiang Zhao, Xiaoli Wei*, Hélder A. Santos* and Xiaofeng Ye**

Supporting Information

Autologous skin fibroblast-based PLGA nanoparticles for treating multiorgan fibrosis

Qiang Long, Zehua Liu, Qianwen Shao, Hongpeng Shi, Shixing Huang, Chenyu Jiang, Bei Qian, Yiming Zhong, Xiaojun He, Xiaogang Xiang, Yang Yang, Bing Li, Xiaoxiang Yan, Qiang Zhao, Xiaoli Wei*, Hélder A. Santos*, Xiaofeng Ye**

[*] Q. Long, H. Shi, S. Huang, C. Jiang, B. Qian, Y. Zhong, X. He, Q. Zhao, X. Ye
Department of Cardiovascular Surgery, Ruijin Hospital, Shanghai Jiao Tong University School of Medicine, Shanghai, 200000, China.
E-mail: xiaofengye@hotmail.com, zq11607@rjh.com.cn

[*] Z. Liu, H. A. Santos
Department of Biomedical Engineering and W.J. Kolff Institute for Biomedical Engineering and Materials Science, University Medical Center Groningen/University of Groningen, Ant. Deusinglaan 1, 9713 AV Groningen, Netherlands.
E-mail: h.a.santos@umcg.nl

Z. Liu, H. A. Santos
Drug Research Program, Division of Pharmaceutical Chemistry and Technology, Faculty of Pharmacy, University of Helsinki, FI-00014 Helsinki, Finland.

[*] Q. Shao, X. Wei
Department of Pharmacology, School of Basic Medical Sciences, Fudan University, Shanghai,
200000, China.
E-mail: xlwei@fudan.edu.cn

X. Xiang
Department of Infectious Diseases, Ruijin Hospital, Shanghai Jiao Tong University School of Medicine, Shanghai, 200000, China.

Y. Yang
Department of Thoracic Surgery, Shanghai Pulmonary Hospital, School of Medicine, Tongji University, Shanghai, 200000, China.

B. Li
Department of Respiratory and Critical Care Medicine, Shanghai Pulmonary Hospital, School of Medicine, Tongji University, Shanghai, 200000, China.

X. Yan
Department of Cardiovascular Medicine, Ruijin Hospital, Shanghai Jiao Tong University School of Medicine, Shanghai, 200000, China.

Keywords: nanoparticle; fibrosis; profibrotic cytokine; myofibroblast

Experimental Section

Fabrication and characterization of FNPs and RNPs. Skin fibroblasts were cultured in 10cm culture dishes, and ~5 million cells per dish were scraped into PBS and pelleted at 700g for 5 minutes when the cells were ~90% confluent. Cells were rinsed in PBS and mechanically disrupted using Dounce homogenizer in the presence of protease inhibitor cocktail. Cell membranes were extracted from the homogenate by differential centrifugation using a Beckman Coulter Optima L-90K Ultracentrifuge as previously described[1]. Homogenate was pelleted for 25 minutes at 10000g, then the supernatant was pelleted for 35 minutes at 150000g. The pellet was resuspended and washed in water containing 0.2 × 10−3 M EDTA. To isolate red blood cells (RBCs) membrane, whole blood was extracted from C57BL/6 male mice through heart puncture with a syringe containing heparin solution. The whole blood was centrifuged at 800g for 10min to separate RBCs from the serum and white blood cells. The packed RBCs were then suspended for 30 minutes at 4°C in lysis buffer (S371KJ, BasalMedia). Resulting lysates were centrifuged twice at 10000g for 5min to remove the hemoglobin and resuspended in water containing 0.2 × 10−3 M EDTA. Cell membranes were stored at -80℃ until use. A bicinchoninic acid test kit was used to measure membrane protein concentrations.

Polymeric cores were synthesized using nanoprecipitation method. 10 mg acid terminated 50:50 Poly(D,L-lactide-co-glycolide)（Mw 24,000-38,000 ,Resomer® RG 503 H） was first dissolved in 1 ml acetone. The acetone solution was then added rapidly to 2 ml ddH2O. To evaporate the organic solvent, the mixture was put in a fume hood for 2 hours. To make fluorescently-labeled polymeric cores, DiD was added to PLGA acetone solution at 0.1 wt-% during the polymer Synthesis. FNPs or RNPs were then made by mixing the cell membranes and Polymeric cores in a disposable sizing cuvette. The mixture was then sonicated for 2 minutes at a frequency of 40 KHz and a power of 200 W using a bath sonicator (KQ-500DE, Kunshan ultrasonic instruments). The hydrodynamic size, surface zeta-potential and dispersity polymer index of nanoparticles were measured using Zetasizer Lab (Malvern Panalytical). The morphology of nanoparticles was examined by transmission electron microscopy (TEM).

Cell isolation and culture. All cells mentioned in this study were cultured at the condition of 37℃ and 5% CO2. To isolate skin fibroblasts or lung fibroblasts, 1 cm of tail tip or distal lung tissue was first obtained from the mouse and minced into fragments of approximately 1 mm2 and immersed with fetal bovine serum. The tissue fragments were then placed on 6-cm culture dishes at an interval of ~0.5 mm and transferred to incubator for 1 h. 2 ml of Dulbecco's

modified Eagle's medium (DMEM) (BasalMedia) supplemented with 10% fetal bovine serum (Gibco) and 1% Penicillin/Streptomycin was then added gently to the culture dish to avoid the floatation of tail fragments. The culture medium was renewed every day and fibroblasts would crawl out of the fragments and attach to the dish (Fig. S23). Skin and lung fibroblasts display spindle-shaped appearances and are positive for FSP-1 staining.

To isolate cardiac fibroblasts, hearts were excised from 5- to -7 d old C57BL/6J neonatal mice, and the ventricles and septum were isolated and minced into small pieces. The heart fragments were then digested using Hank's balanced salt solution (HBSS) solution containing 1 mg ml-1 type II collagenase (Basal media) and incubated at 37℃ under moderate stirring for ~40 min. The cell suspension was then filtered through 70 μm cell strainer and centrifuged at 300g for 5 min. The cell pellet was resuspended with complete medium and transferred to 6-cm culture dishes. After 30 min, the cardiac fibroblasts would attach to the dishes and the culture medium was renewed to remove the supernatant cardiomyocytes and dead cells. Cardiac fibroblasts display spindle-shaped appearances and are positive for FSP-1 staining.

To isolate aortic endothelial cells[2], mouse was firstly anesthetized by inhalation of 2% isoflurane, the chest was then opened, five milliliters of PBS containing 1,000 U/mL of heparin was injected to the left ventricle to perfuse the aorta. Afterwards, aorta was excised and cut into 1 mm rings using a sterile scalpel blade. The aorta rings were then cut open by microscissors and placed lumen-side-down on the growth factor-reduced matrix-coated dish. Moderate endothelial cell growth medium (Sciencell) was added to the dish to immerse the aorta pieces. The culture medium was renewed every day and endothelial cells would crawl out of the aorta pieces and attach to the dish. Endothelial cells display cobblestone-like appearances and are positive for CD31 staining.

NMuMG cells were purchased from ATCC and maintained in DMEM supplemented with 10% fetal bovine serum, 2 mM L-glutamine, 1% Penicillin/Streptomycin and insulin (10 μg ml-1). HSC-T6 cell line and Raw264.7 cell Line were purchased from Shanghai Zhong Qiao Xin Zhou Biotechnology and maintained in DMEM supplemented with 10% fetal bovine serum and 1% Penicillin/Streptomycin. Every 1-2 days, the medium was renewed. Cells were passaged using trypsin when they reached ~90% confluence[3].

In vitro cellular uptake of bare PLGA nanoparticles and FNPs. The cellular uptake study was conducted in RAW 264.7 cells and mouse primary cardiac fibroblasts. Before the experiment, equal numbers of cells were planted on glass coverslips and fasted in serum-free media for 24 hours. At a final concentration of 0.1 mg ml^{-1}, DiD-labeled FNPs or bare PLGA

particles were added to the culture medium and incubated with FNPs or bare PLGA nanoparticles for 4 hours. After that, the cells were rinsed three times in PBS and fixed for 10 minutes in a 4% formaldehyde solution. Cells were then rinsed for more three times in PBS and stained by Atto 488 Phalloidin (Sigma, 49409) for 30 min. The coverslips were then mounted by mounting medium with DAPI (ab104139, abcam). A confocal laser scanning microscope (LSM880, Zeiss) was used to image the cells.

Profibrotic cytokine binding test. Recombinant mouse TGF-β1 (7666-MB, R&D systems), IL-11 (418-ML, R&D systems), IL-13 (413-ML, R&D systems), IL-17 (421-ML, R&D systems) were mixed with FNPs or RNPs at final concentrations ranging from 0-2 mg ml^{-1}. the mixtures were incubated at 37°C for 2 h and then centrifuged at 14000g for 15min to pellet the nanoparticles. Concentrations of cytokines in the supernatant were measured using mouse TGF-β1, IL-11, IL-13, IL-17 Elisa kit (Solarbio). To examine the cytokine binding capacity of AH, FNP-AH and RNP-AH, mouse TGF-β1, IL-11, IL-13, IL-17 were incubated with the hydrogels (final FNPs/RNPs concentrations: ~1 mg ml^{-1}) at 37°C for 2 h, concentrations of cytokines in the supernatant were measured using ELISA kits.

TGF-β1-induced myofibroblast differentiation assay. Mouse primary lung fibroblasts, cardiac fibroblasts, hepatic stellate cells, NMumG cells and aortic endothelial cells were seeded in 6-well culture plates. For immunofluorescent imaging experiments, cells were seeded on glass coverslips. When the cells were ~50% confluent, cells were starved for 24 h in serum-free medium. Subsequently, TGF-β1 (final concentrations: 5 ng ml^{-1}) and FNPs/RNPs (final concentrations: 1 mg ml^{-1}) were added to the complete culture medium. Cells were incubated at 37°C for 24 h.

Collagen gel contraction assay. Collagen gel contraction assays were performed using mouse primary lung fibroblasts using a CytoSelect™ 24-Well Cell Contraction Assay Kit (Floating Matrix Model) (CBA-5020, Cell Biolabs) as per the manufacturer's protocol. Briefly speaking, cells suspended in complete media (1×10^6 cells ml^{-1}) were used to create a 1:4 mixture with collagen gel working solution. 500 ul of solution were added to each well of the 24-well cell contraction plate. Following 1 h of polymerization, complete culture media was added atop each collagen gel lattice. TGF-β1 (final concentrations: 5 ng ml^{-1}) and FNP/RNP (final concentrations: 1 mg ml^{-1}) were added to the culture medium. Gels were

photographed every 24 hours for 48 hours. Gel area was measured after 48 hours using ImageJ (NIH) and normalized to original gel area.

Cell migration assay. The migration ability of mouse primary lung fibroblasts was determined using Boyden chamber assays. Cells were prepared as described above. Mouse primary lung fibroblasts were seeded in 6 well culture plate. When the cells were ~50% confluent, cells were starved for 24h in serum-free medium. TGF-β1 (final concentrations: 5 ng ml^{-1}) and FNP/RNP (final concentrations: 1 mg ml^{-1}) were added to the culture media. Cells were incubated at 37°C for 24 h. Subsequently, Cells were washed with PBS for 3 times. The Cells were then detached using 0.25% trypsin-EDTA, pelleted at 400g for 5min and resuspended at a density of 2.5×10^5 cells ml^{-1} using serum free culture media. 200 µl of the cell suspensions (~5×10^4 cells) were added to the trans-well insert, 800 µl of complete culture media was added to the lower chamber. After an 8-hour incubation period, the cells were fixed in methanol and stained for 30 minutes at room temperature with a 1% crystal violet solution. Cotton swabs were used to remove non-migratory cells. Migratory cells were photographed and counted from three non-overlapping fields per membrane.

Immunoblotting. Tissue samples were homogenized and lysed by RIPA lysis buffer (P0013B, Beyotime) containing protease and phosphatase inhibitor cocktail (P1045, Beyotime). Protein concentrations were quantified by bicinchoninic acid assay (ZJ102, EpiZyme). The protein solutions were diluted by 4X LDS Sample Buffer (Biofuraw™) and heated at 95°C for 10min. Cell samples were lysed directly by 1X SDS-PAGE Sample Loading Buffer (P0015A, Beyotime) containing PMSF and heated at 95°C for 10min. Equal amount of protein was submitted to gel electrophoresis on 4-20% bis-tris gels (180-8005HB, Biofuraw™) in Mops running buffer (BT8100, Biofuraw™) and then transferred to polyvinylidene difluoride membranes (IPVH00010, Millipore) in transfer buffer (8006-6006, Biofuraw™). Membranes were blocked (P0252FT, Beyotime) and incubated with primary antibodies overnight at 4°C. The membranes were washed and incubated with secondary antibodies for 1h at room temperature. All antibodies were listed in **Table S2**. Protein bands were visualized using Tanon 4200 image Analyzer and quantified using the ImageJ Software (NIH, version 1.52).

qPCR assay. Total RNA was extracted from cells using MolPure® Cell/Tissue Total RNA Kit (19221ES, Yeasen Biotech) according to manufacturer's instructions. The cDNA was

prepared using Hifair® III 1st Strand cDNA Synthesis Kit (11139ES, Yeason Biotech). qPCR was performed using Hieff UNICON® Universal Blue qPCR SYBR Green Master Mix (11184ES, Yeasen Biotech) and analyzed using Light cycler 480 (Roche). All primers for qPCR reactions were listed in **Table S3**. Gene expression fold change was calculated using $2^{-\Delta\Delta Ct}$ method normalized to GAPDH.

Animal study ethics

Male C57BL/6J mice (6-8 weeks) were purchased from Zhejiang Charles River Laboratories and housed at a constant temperature (22 ± 2 °C) under a 12-h light/dark cycle and given standard lab chow and water ad libitum. At the end of the experiments, mice were euthanized by carbon dioxide inhalation. All animal experiments were carried out in accordance with guidelines evaluated and approved by the Animal Experimental Ethics Committee of Shanghai Jiao Tong University (Approval no. 10903).

Bleomycin-induced lung fibrosis and FNP/RNP delivery. 8-week-old C57BL/6 mice were anesthetized with isoflurane before the exposure of trachea. Lung fibrosis was induced with bleomycin (50 µl at 3.5 mg kg^{-1}) (HY-17565A, MCE) delivered intratracheally to the lungs using Micro-Sprayer Aerosolizer (YSKD biotechnology, Beijing) at day 0[4]. From day 3, mice received i.t. administration of FNPs, RNPs (50 µl at 2 mg ml^{-1}) via a micro-sprayer every 5 days till day 21 (**Movie S1**). The vehicle-treated mice received sterile PBS solution instead, using identical methods.

CCl_4-induced liver fibrosis. To induce liver fibrosis, 8-week-old C57BL/6 mice received intraperitoneal injection of CCl_4/mineral oil (7:1, v/v) at a dose of 1 µl g^{-1} twice weekly for 6weeks[5]. Mice were administered therapeutic treatment with FNPs or RNPs (100 µl at 2 mg ml^{-1}) by tail vein injection once weekly for 6 weeks. At the end of observation (72 h after the final injection of CCl_4), mice were euthanized. Whole blood was collected by heart puncture using a syringe containing heparin solution. ALT and AST levels were tested using serum isolated from the whole blood.

Fabrication and characterization of AH, FNP-AH and RNP-AH. Sodium alginate powder (180947, Sigma) and calcium alginate powder (21054, Sigma) were dissolved in in sterilized water to form 2% (wt/vol) sodium alginate solution and 1.5% (wt/vol) calcium alginate suspension respectively. FNPs/RNPs (final concentration: 4 mg ml^{-1}) were added to the

sodium alginate solution and then crosslinked with calcium alginate suspension to prepare the hydrogel composite (**Fig. S24**). The morphology of the FNP-AH was observed by scanning electron microscopy. The rheological properties of FNP-AH were tested using a HAAKE MARS III rheometer (Thermo Scientific).

MI-induced cardiac fibrosis. To induce cardiac fibrosis, permanent ligation of the left anterior descending coronary artery was performed on 8-week-old C57BL/6 mice[6]. Mice were anesthetized by isoflurane and then intubated and subjected to mechanical ventilation. The chest was opened via left thoracotomy at the 2nd intercostal space and heart was exposed. The pericardium was removed and the LAD was permanently ligated by 8-0 silk suture (**Movie S2**). Sham operated animals were subjected to the same procedures without aorta ligation. ~20 µl of AH, FNP-AH or RNP-AH was injected over 4 regions of the infarcted area (~5 µl per injection). To prevent entering the ventricular chamber, injections were given with the needle parallel to the ventricle's long axis (**Movie S3**).

Ultrasound imaging. Transthoracic echocardiography was performed with a Vevo 3100 instrument (FUJIFILM VisualSonics) equipped with an MX-550D imaging transducer as previously described[7]. Mice were slightly anesthetized with isoflurane and the chest hair was removed. During the echocardiographic examination, heart rate was maintained at approximately 500 bpm in all mice. Echocardiographic data including LVESD, LVEDD, LVESV, LVEDV, LVEF and LVFS by M-mode assessments were collected and analyzed on day 7 and day 28. The ultrasonography of liver was performed using the same instrument on Week 0, week 2, week 4 and week 6 as previously described[5]. Mice were anesthetized with isoflurane and the abdomen fur was removed. The livers were scanned in the parasternal long axis view and the portal vein area was measured three times. Image J (1.52v, NIH) was used to quantify the brightness of each ultrasound image.

lung function test. The parameters of lung function were determined utilizing an invasive pulmonary function testing equipment (shanghai TOW intelligent technology). The mice were first anesthetized by isoflurane, then an endotracheal cannula was inserted into the trachea and connected to the testing system. FVC, dynamic lung compliance, FEV100, FEV 300, FEV 300, expiratory reserve volume (ERV), peak expiratory flow (PEF) and lung resistance (RL) were obtained from the system.

Bronchoalveolar lavage of mice lungs. Mice were firstly anesthetized by 2% isoflurane. The trachea was exposed and a lavage catheter was inserted into the trachea. The catheter was stabilized by tying the trachea around the catheter using 3-0 silk thread. A 1 mL syringe was connected to the catheter and 1 ml of cold PBS (0.5 ml each, for 2 time) was injected into the lung. The solution was then gently aspirated from the mouse lung[8]. ~0.7 ml of bronchoalveolar lavage fluid (BALF) was collected and centrifuged at 300g for 5 min. Concentrations of TGF-β1 in the supernatant were measured using a mouse TGF-β1 Elisa kit (Solarbio).

BrdU incorporation assays. To measure lung fibroblast proliferation, cells were seeded on glass coverslips in a six well dish. Cells were incubated with 50 μM BrdU (B8010, Solarbio) for two hours and fixed in 4% paraformaldehyde for 10 min at room temperature. Cell membranes were permeabilized using 0.5% Triton X-100 for 10 min followed by nuclear permeabilization using 2 N HCl for 30 min and stained with anti-BrdU antibody overnight.

SHG imaging. SHG images of collagen fibers were acquired using a multiphoto laser scanning microscopy (Olympus FVMPE-RS). Each image was integrated for 7 microseconds/pixel and recorded using a 25x water immersion objective (Olympus UMPLFLN, N.A. 1.05). SHG signals were generated using 860 nm picosecond laser and collected using 400±40 nm filter. Skewness (asymmetry of pixel distribution) and kurtosis (gray-tone spread-out distribution) were assessed using image J as indicators of fiber arrangement[9].

Atomic Force Microscopy (AFM). The Young's modulus of tissues was determined using an AFM (FastScan-bio, Bruker, CA). Fresh scar tissue from the infarcted heart was cut into approximately 3 x 3 mm^2 fragments. Tissue fragments were adhered equally to a culture plate and immersed in PBS solution. The Young's modulus of tissues was determined using an AFM tip with a diameter of 5 μm. The Young's modulus values were determined using NanoScope analysis (1.7v, Bruker) from each sample's force-displacement curves.

^{18}F-FDG PET–CT imaging and pulmonary CT scanning. The ^{18}F-FDG assessments were performed on a small animal PET scanner (Inveon, Siemens) as previously described[6a]. Forty-five minutes after intraperitoneal administration of approximately 120 μCi ^{18}F-FDG, CT images were acquired for 5 min and PET images were acquired for 15 min. Mice were

anesthetized with 2% isoflurane throughout the procedure. The images of PET and CT were acquired, reconstructed and fused using Inveon Research Workplace (Siemens Healthcare, Germany). Seventeen-segment polar map was constructed using Carimas 2.10 (Turku PET center, Finland). The infaction size was calculated by the following formula: infarction % = (area with SUV lower than 50% of the max SUV of the whole heart / total heart area) × 100%. For pulmonary CT scanning, Mice were anesthetized with 2% isoflurane and CT images were acquired for 10 min.

Histological assessment. After 24 hours of fixation in 4% formalin, the tissues were embedded in paraffin and sectioned for histological staining. Histological images were obtained using a Pannoramic Digital Slide Scanner (MIDI II, 3D HISTECH). For histological analysis of lung fibrosis, H&E staining was conducted. All H&E stained slides were scanned at 20× magnification and 8 non-overlapping fields were scored. The severity lung fibrosis was estimated on a numerical scale according to Ashcroft et al[10].The mean of all scores was calculated for each slide. For histological analysis of liver fibrosis and cardiac fibrosis, Masson's trichrome staining was conducted. Collagen volume fraction was quantified using Image J. the fibrotic size% of heart was calculated by the following formula: fibrotic size% = (total infarct circumference/total LV circumference) × 100%, as previously described[11].

In vivo fluorescence imaging. To study the biodistribution of FNPs/RNPs, FNPs and RNPs were labeled with 1,1′-dioctadecyl-3,3,3′,3′-tetramethylindotricarbocyanine iodide (DiR, 0.1wt%, excitation = 748 nm/emission = 780 nm). Mice received intratracheal or intravenous administration of FNPs/ RNPs (50 µl at 2 mg ml^{-1}). After 24 h, mice were sacrificed. the major organs including lung, heart, liver, spleen and kidney were harvested and imaged using IVIS Spectrum CT (In Vivo Imaging System, PerkinElmer). The average radiant efficiency of each organ was analyzed by Living Image 4.5.4 Software (In Vivo Imaging System, PerkinElmer). To study the retention of FNPs/RNPs in the lung, bleomycin-treated mice received intratracheal administration of FNPs/RNPs (50 µl at 2 mg ml^{-1}). Mice were sacrificed and the lungs were harvested and imaged on day 0, day 1, day 2, day 3, day 4, day 5, day 7 and day 14. To study the retention of FNPs/RNPs in the liver, CCl4-treated mice received intravenous administration of FNPs/RNPs (100 µl at 2 mg ml^{-1}). The abdomen fur was removed, and mice were imaged and analyzed on days 0, 1, 3, 5, 7, 9 and 14. To study the retention of FNP-AH/RNP-AH in the heart, mice received intramyocardial injection of 20

μl FNP-AH/RNP-AH. Mice were sacrificed and the heart were harvested and imaged on days 0, 7, 14 and 21.

Hydroxyproline assays. The total collagen content of freshly extracted lung and liver tissue was evaluated using the Hydroxyproline Assay Kit (BC0255, Solarbio) and adjusted to the total tissue weight per the manufacturer's protocol.

RNA sequencing analysis. Total RNA was isolated from each sample independently using the Trizol reagent (Invitrogen). The RNA quality was determined using an Agilent 2200 and stored at 80°C. The cDNA libraries for each RNA sample were created according to the manufacturer's protocols using the TruSeq Stranded mRNA Library Prep Kit (Illumina, Inc.). The libraries were quality-controlled using an Agilent 2200 and sequenced on a 150-bp paired-end run with a Hiseq XTen. Using the Hisat2 software, the clean reads were aligned to the mouse genome (GRCm38, NCBI). Gene counts were obtained using HTseq[12], and gene expression was determined using the RPKM technique. Gene set enrichment analysis (GSEA) was performed using GSEA version 4.1.0 software (Broad Institute) as previously described[13]. The statistical significance was assessed using 10,000 random permutations of the gene set with a signal-to-noise metric for ranking genes. A FDR-corrected value of $q < 0.25$ was considered to be significant. Gene sets were obtained from the Molecular Signatures Database (MSigDB) version 7.4.

Proteomic analysis. SDT (4% SDS, 100mM Tris-HCl, 1mM DTT) buffer was used to extract total proteins from the samples, which were then digested with trypsin using the filter-aided sample preparation (FASP) method. Each sample's digest peptides were desalted using C18 Cartridges (Sigma), vacuum centrifuged, and reconstituted in 40 μl of 0.1%(v/v) formic acid. TMT reagent was used to label 100 ug of each sample's peptide mixture according to the manufacturer’s protocols (Thermo Scientific). The High pH Reversed-Phase Peptide Fractionation Kit was used to fractionate the labelled peptides (Thermo Scientific). For 60/90 minutes, LC-MS/MS analysis was carried out using a Q Exactive mass spectrometer (Thermo Scientific) linked to Easy nLC (Thermo Fisher Scientific). For identification and quantification analysis, the MASCOT engine (Matrix Science, London, UK; version 2.2) included in Proteome Discoverer 1.4 software was used to search the MS raw data for each sample.

Statistical analysis. Data are presented as bar plots (mean ± s.d.) or box-and-whisker plots, indicating the median value (black bar inside box), 25th and 75th percentiles (bottom and top of box, respectively), and minimum and maximum values (bottom and top whisker, respectively). Exact n values for all experiments are presented in the figure legends or inside the figure itself. Differences between two groups were analyzed by a two-tailed Student's *t*-test. For comparisons between more than two groups, a one-way ANOVA with Tukey's post hoc test was performed. A Kaplan–Meier curve was created to present the cumulative survival and differences in cumulative survival were tested by the log-rank test using IBM SPSS Statistics (Version 23.0). For all comparisons, $p < 0.05$ was considered significant.

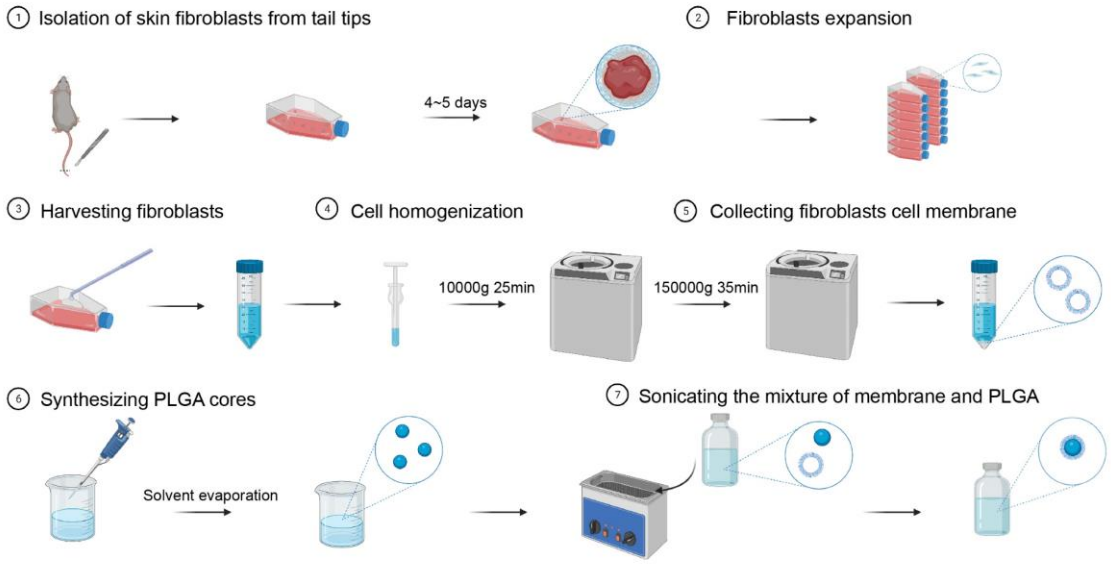

Figure S1. Schematic representation for fabrication of FNPs.

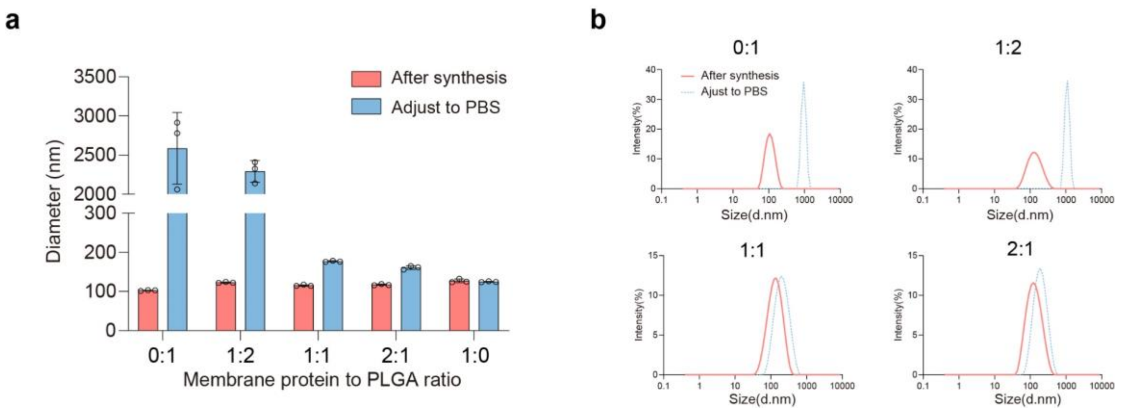

Figure S2. Optimization of membrane protein-to-polymer weight ratios. (**a**) Hydrodynamic size of FNPs prepared with various membrane protein-to-polymer weight ratios after sonication and adjusting the solution to 1X PBS. (**b**) Size distribution curves of FNPs prepared with various membrane protein-to-polymer weight ratios after sonication and adjusting the solution to 1X PBS. Data are expressed as mean ± s.d.

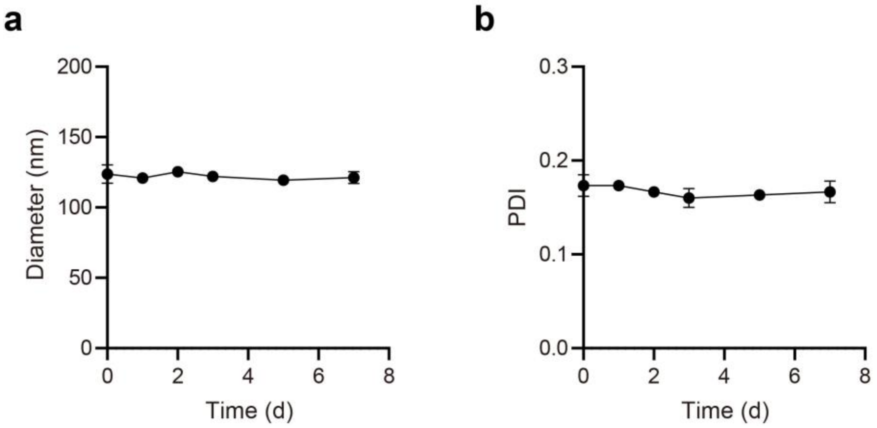

Figure S3. Stability of FNPs. Mesasurements of (**a**) hydrodynamic size and (**b**) PDI of FNPs stored in 1X PBS at 4℃ for 7 days (n = 3 independent experiments).

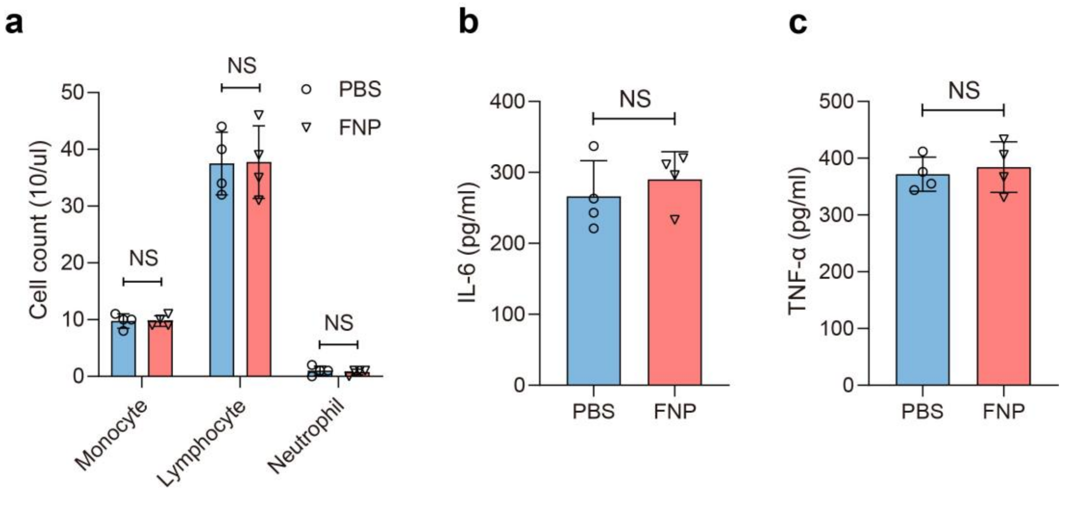

Figure S4. Immune cell count and levels of proinflammatory cytokines in the plasma. (**a**) Cell count of monocytes, lymphocytes, neutrophils and levels of (**b**) IL-6, (**c**) TNF-α in the plasma measured 24 hours after intravenous injection of FNPs (20 mg kg^{-1}) or PBS.

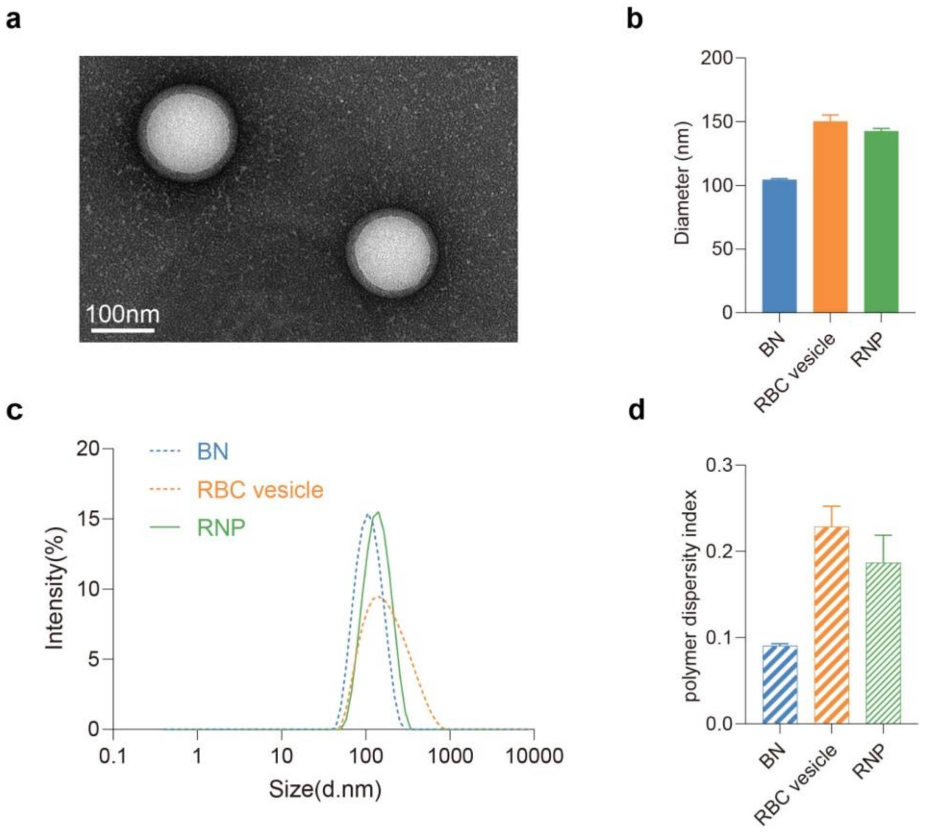

Figure S5. Characterization of RNPs. (**a**) Transmission electron microscopy images of RNPs negatively stained with uranyl acetate. (**b**) Hydrodynamic size, (**c**) Size distribution curves, and (**d**) Polymer dispersity index of RNPs. Data are expressed as mean ± s.d.

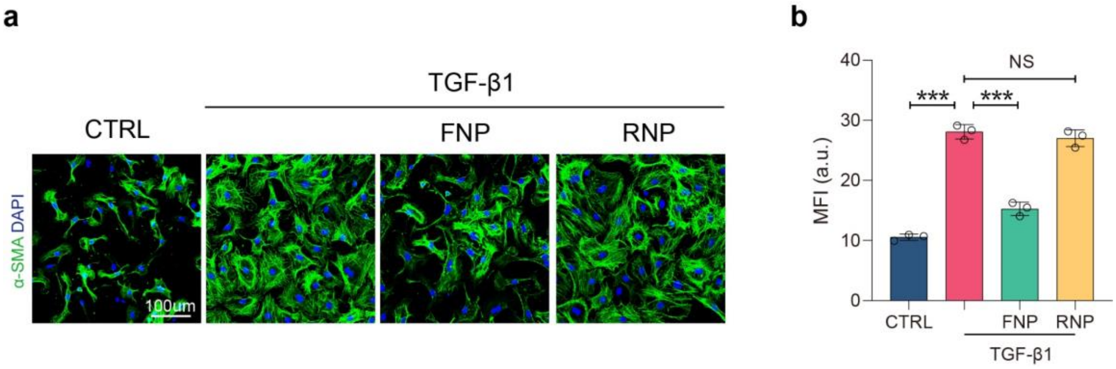

Figure S6. FNPs attenuate TGF-β1-induced cardiac fibroblast to myofibroblast differentiation. (**a**-**b**) Mouse primary cardiac fibroblasts were incubated with TGF-β1 (5ng/ml), FNPs, RNPs or without stimulation (CTRL) for 24 hours (n=3 biologically independent samples). (**a**) Representative immunofluorescent images of cardiac fibroblasts showing α-SMA, nuclei were labelled with DAPI. (**b**) Quantification of mean fluorescent intensity of α-SMA. Data are expressed as mean ± s.d. Data were analyzed by one way ANOVA with Tukey's post hoc test, NS indicates not significant, ***$p < 0.001$.

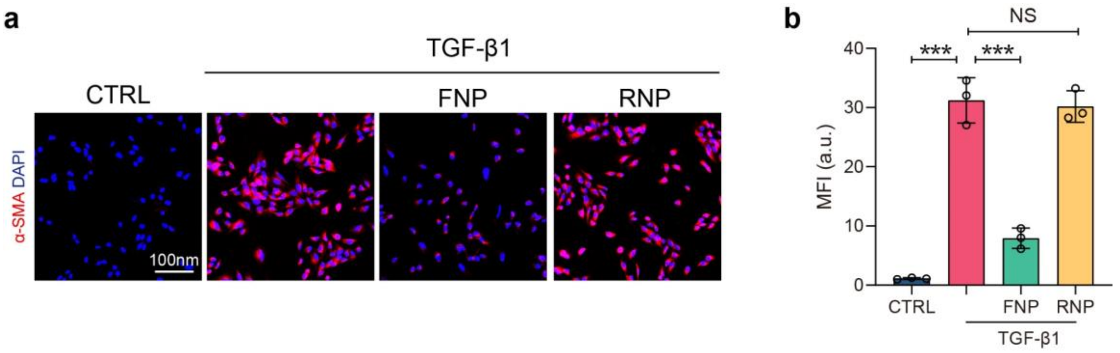

Figure S7. FNPs attenuate TGF-β1-induced hepatic stellate cell to myofibroblast differentiation. (**a-b**) HSC-T6 cells were incubated with TGF-β1 (5ng/ml), FNPs, RNPs or without stimulation (CTRL) for 24 hours (n=3 biologically independent samples). (**a**) Representative immunofluorescent images of HSC-T6 cells showing α-SMA, nuclei were labelled with DAPI. (**b**) Quantification of mean fluorescent intensity of α-SMA. Data are expressed as mean ± s.d. Data were analyzed by one way ANOVA with Tukey's post hoc test, NS indicates not significant, ***$p < 0.001$.

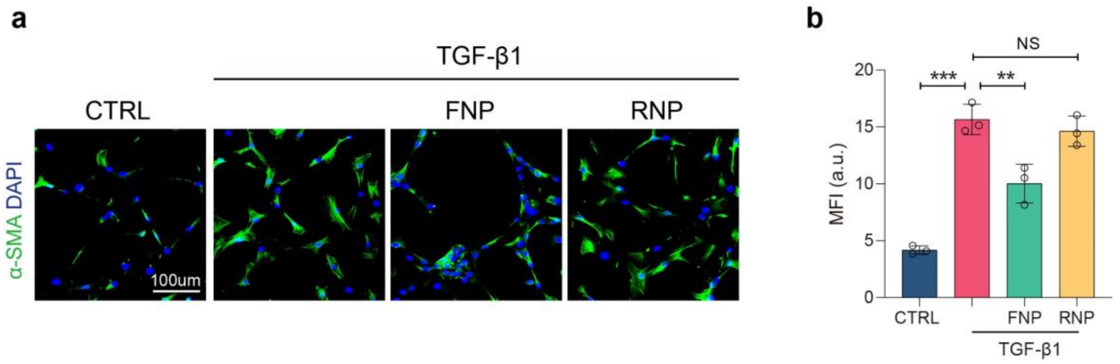

Figure S8. FNPs attenuate TGF-β1-induced endothelial to mesenchymal transition. (**a-b**) Mouse primary aortic endothelial cells (MACEs) were incubated with TGF-β1 (5ng/ml), FNPs, RNPs or without stimulation (CTRL) for 24 hours (n=3 biologically independent samples). (**a**) Representative immunofluorescent images of MACEs showing α-SMA, nuclei were labelled with DAPI. (**b**) Quantification of mean fluorescent intensity of α-SMA. Data are expressed as mean ± s.d. Data were analyzed by one way ANOVA with Tukey's post hoc test, NS indicates not significant, ***$p < 0.001$.

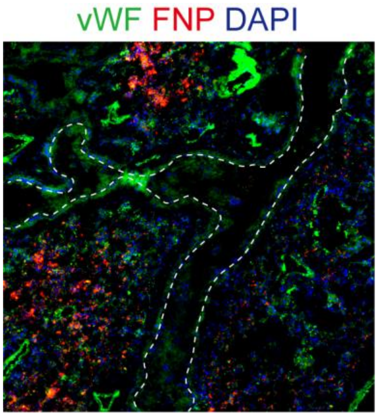

Figure S9. Distribution of FNPs in the lungs from bleomycin-treated mice. FNPs were labeled with DiD (red), dash lines outline the bronchi.

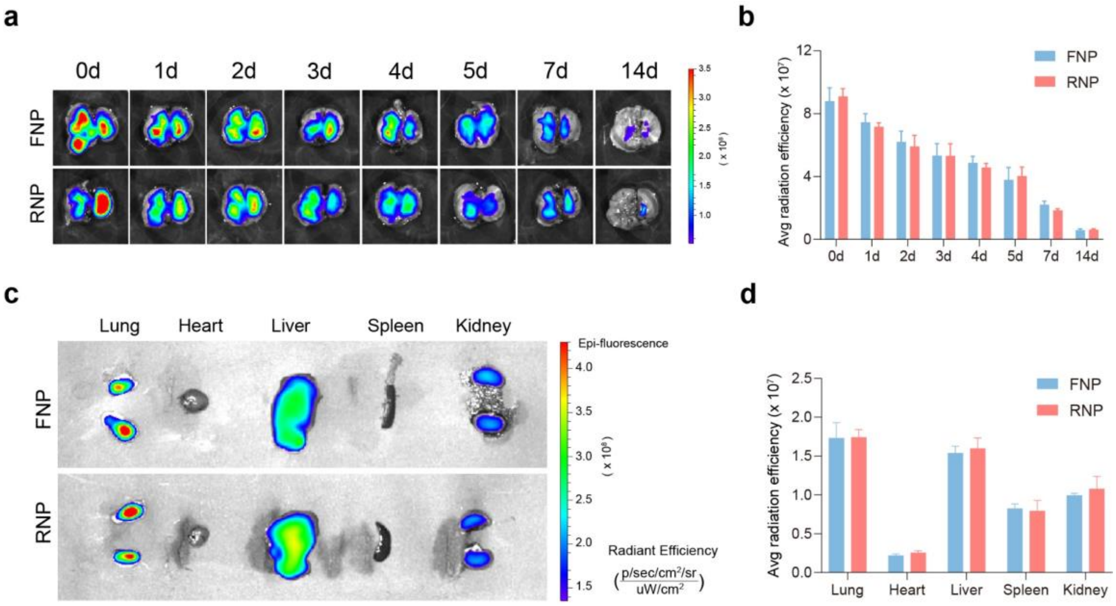

Figure S10. Lung retention and biodistribution of FNPs/RNPs. (**a**) Representative fluorescence images of lungs from bleomycin-treated mice receiving atomization of FNPs or RNPs on day 0, day 1, day 2, day 3, day4, day 5, day 7 and day 14. (**b**) Quantification of the average efficiency as observed in **a** (n=3 biologically independent mice per group). (**c**) Representative fluorescence images of major organs at 24 hours from bleomycin-treated mice receiving atomization of FNPs or RNPs. (**d**) Quantification of the average efficiency as observed in **c** (n=3 biologically independent mice per group). Data are expressed as mean ± s.d.

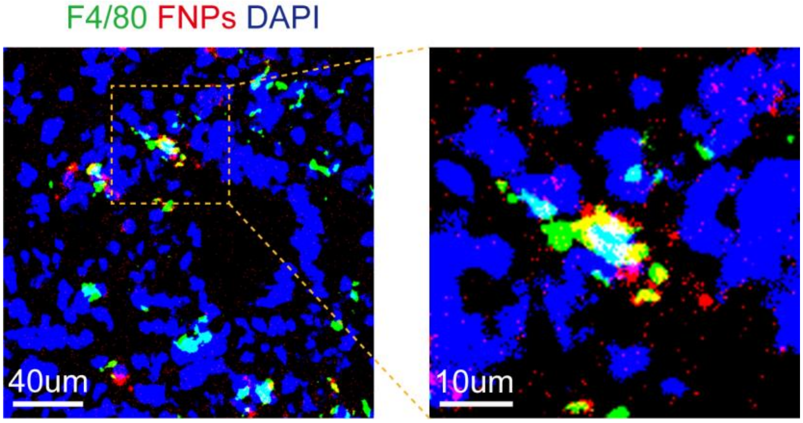

Figure S11. Colocalization of fluorescently labeled FNPs with F4/80^{+} macrophages in bleomycin-treated lungs.

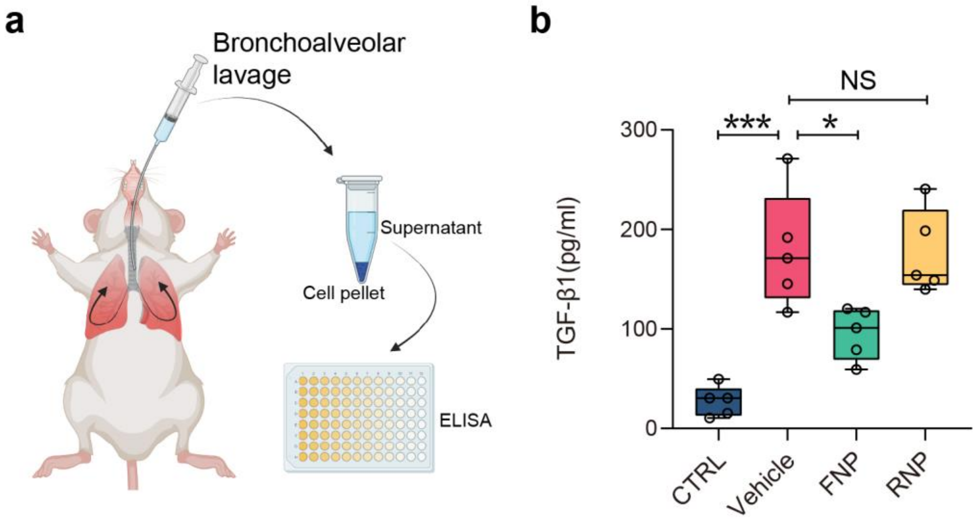

Figure S12. TGF-β1 levels in BALF. (**a**) Schematic representation for measurement of TGF-β1 levels in BALF. (**b**) TGF-β1 levels in BALF for each group. Data are presented as box-and-whisker plots. Data were analyzed by one way ANOVA with Tukey's post hoc test, NS indicates not significant, $*p < 0.05$, $***p < 0.001$.

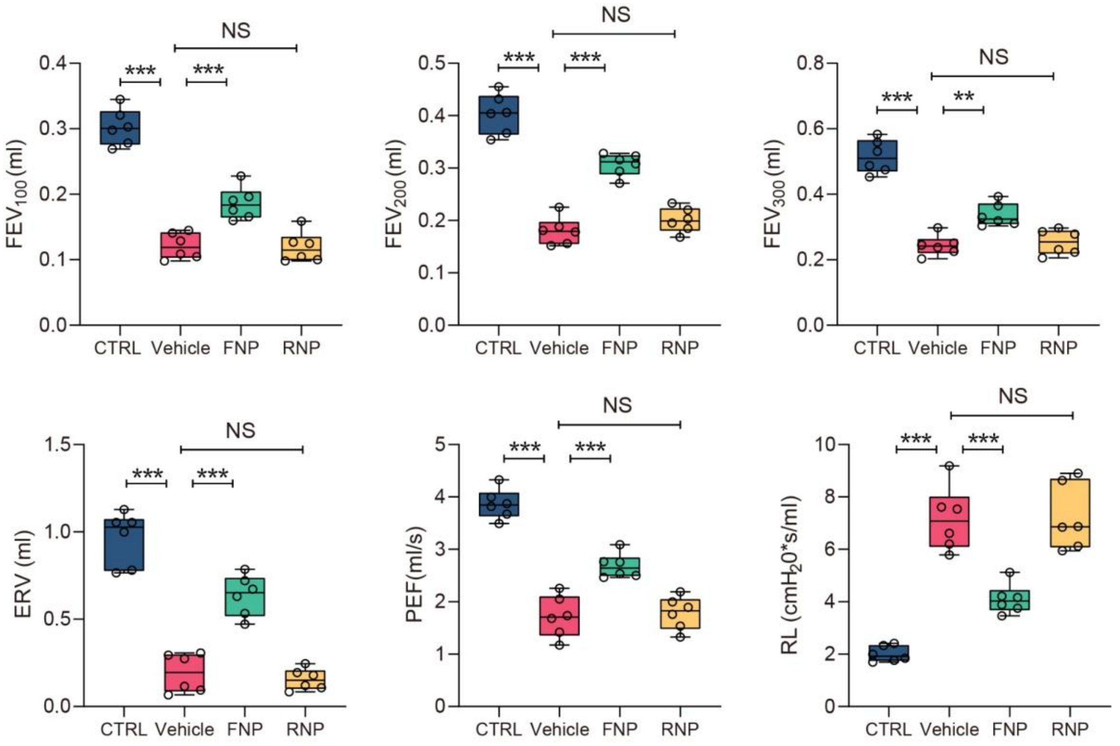

Figure S13. FNP treatment improves lung functions. n=6 biologically independent mice per group. FEV, forced expiratory volume: amount of air exhaled measured during the first 100ms (FEV_{100}), 200ms (FEV_{200}), and 300ms (FEV_{300}) of the forced breath; ERV, expiratory reserve volume; PEF, peak expiratory flow; RL, total lung resistance. Data are presented as box-and-whisker plots. Data were analyzed by one way ANOVA with Tukey's post hoc test, NS indicates not significant, $^{**}p < 0.01$, $^{***}p < 0.001$.

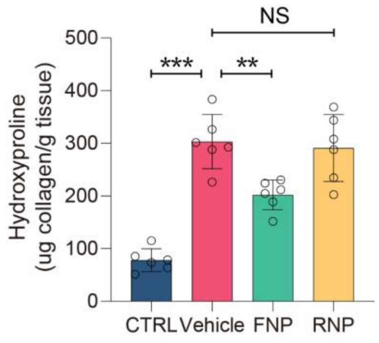

Figure S14. Hydroxyproline content of lungs for each group. n=6 biologically independent mice per group. Data are expressed as mean ± s.d. Data were analyzed by one way ANOVA with Tukey's post hoc test, NS indicates not significant, $^{**}p < 0.01$, $^{***}p < 0.001$.

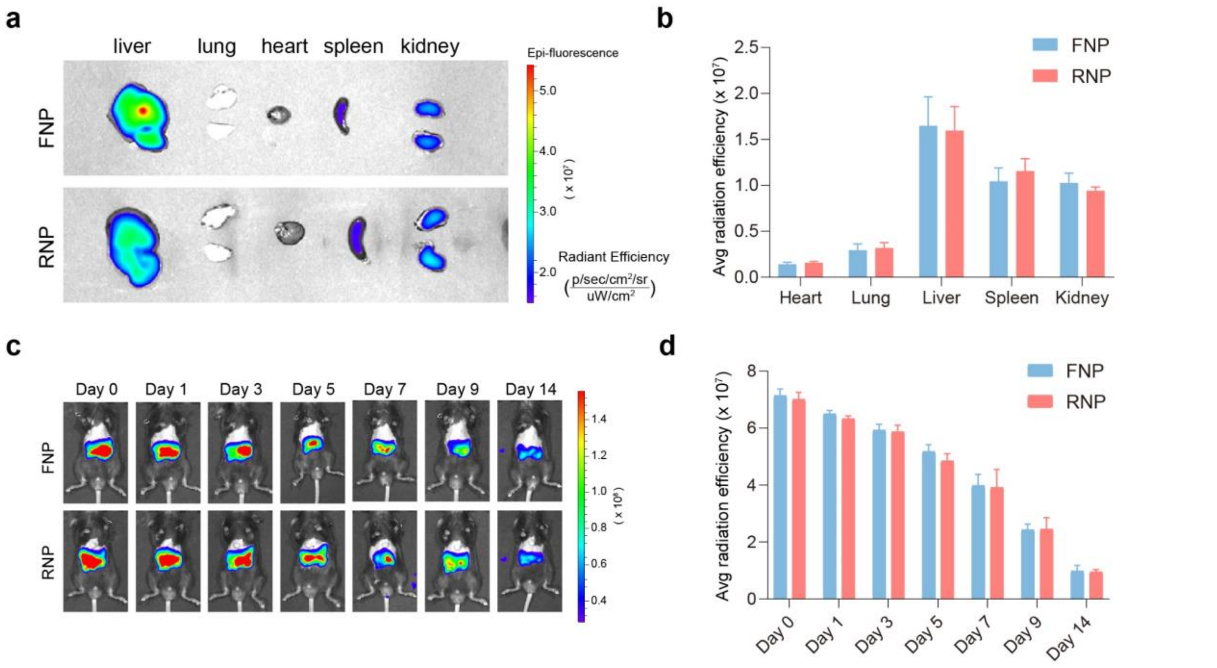

Figure S15. Biodistribution and liver retention of FNPs/RNPs. (**a**) Representative fluorescence images of major organs at 24 hours from CCl_4-treated mice receiving intravenous injection of FNPs or RNPs. (**b**) Quantification of the average efficiency as observed in **a** (n=3 biologically independent mice per group). (**c**) Representative fluorescence images of CCl4-treated mice receiving intravenous injection of FNPs or RNPs on day 0, day 1, day 3, day 5, day7, day 9 and day 14. (**d**) Quantification of the average efficiency as observed in **c** (n=4 biologically independent mice per group).

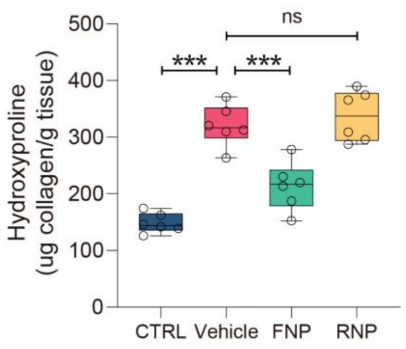

Figure S16. Hydroxyproline content of livers for each group. n=6 biologically independent mice per group. Data are presented as box-and-whisker plots. Data were analyzed by one way ANOVA with Tukey's post hoc test, NS indicates not significant, ***$p < 0.001$.

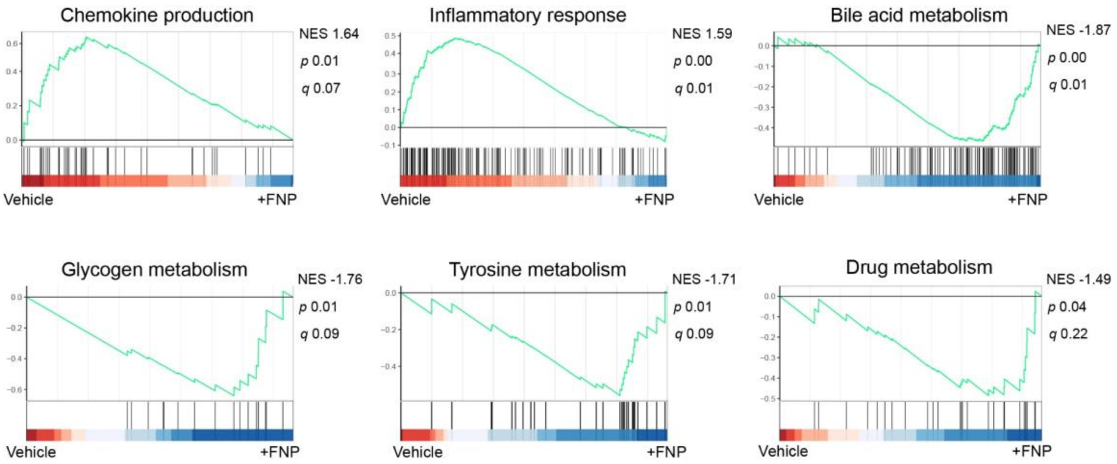

Figure S17. GSEA of liver RNA-seq signals of MSigDB-defined gene clusters.

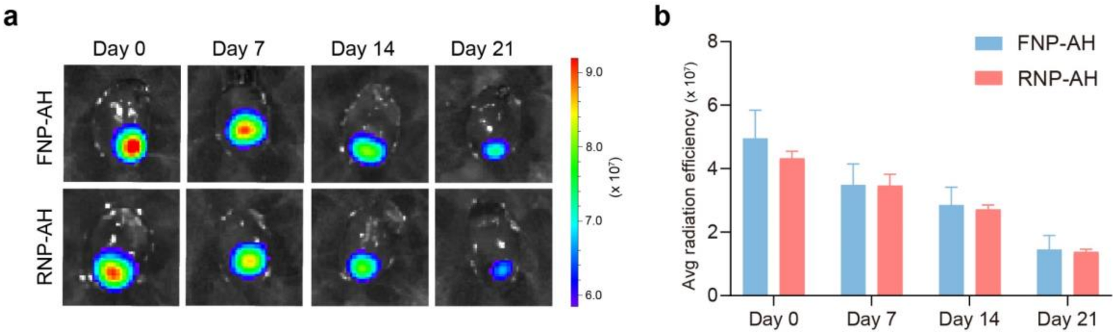

Figure S18. Retention of FNP-AH/RNP-AH in myocardium. (**a**) Representative fluorescence images of heart from mice receiving intramyocardial injection of FNP-AH or RNP-AH on day 0, day 7, day 14, day 21. (**b**) Quantification of the average radiation of efficiency as observed in **a** (n=3 biologically independent mice per group). Data are expressed as mean ± s.d.

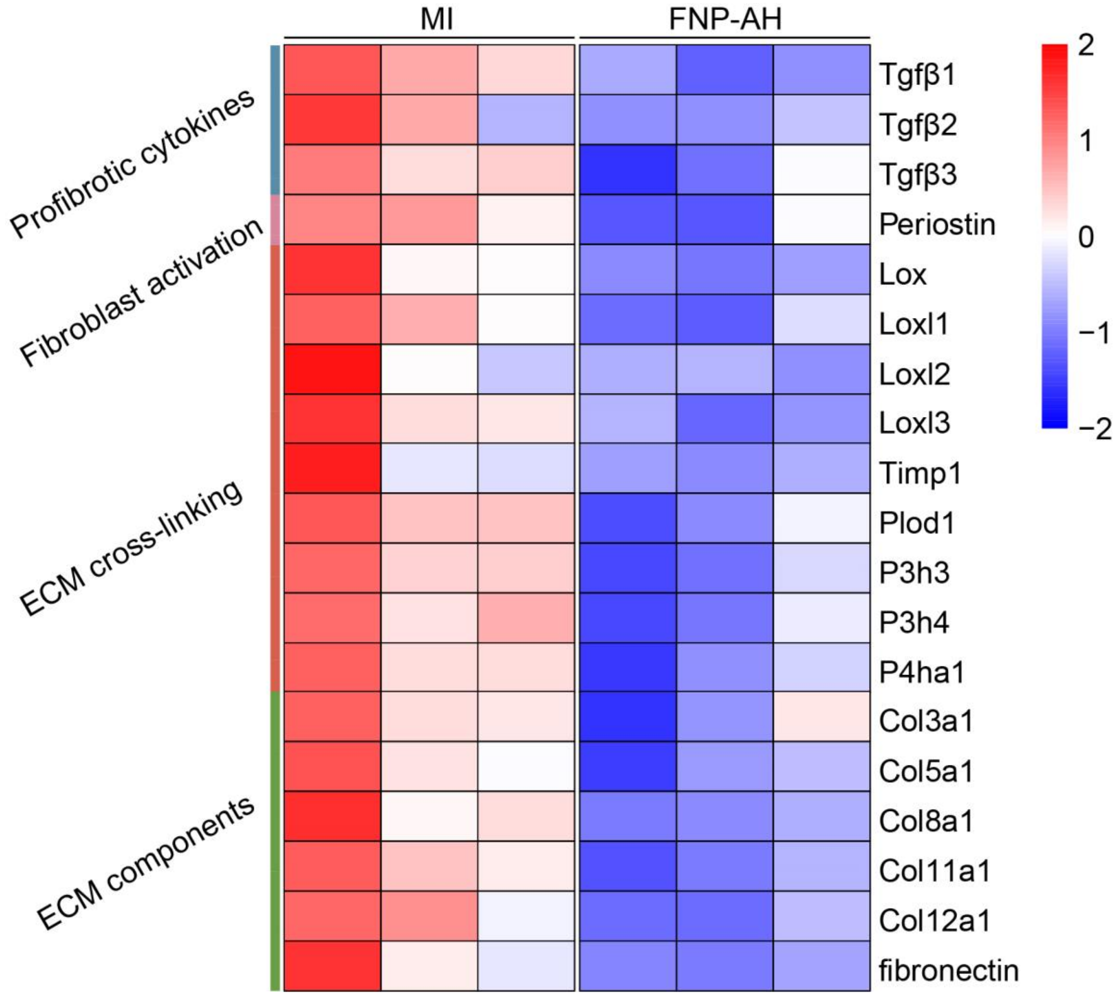

Figure S19. Heatmap of fibrosis-related proteins expression in MI group and FNP-AH group.

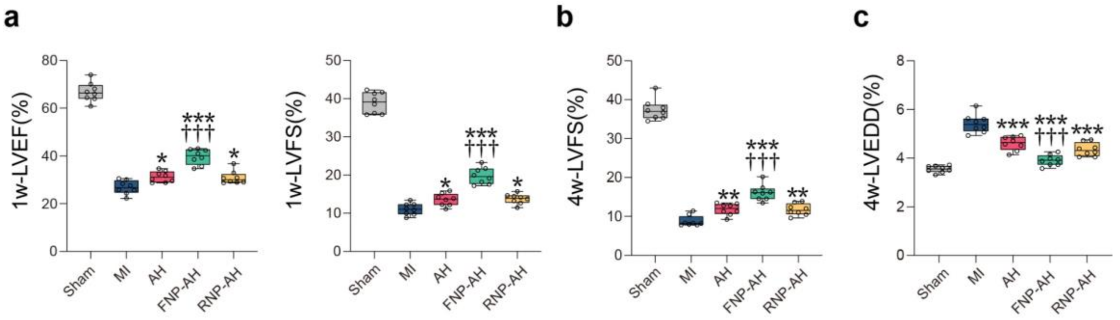

Figure S20. Assessment of heart functions by echocardiography. (**a**) LVEF and LVFS measured on week 1 (n=8 biologically independent mice per group). (**b**) LVFS and (**c**) LVEDD measured on week 4 (n=8 biologically independent mice per group). Data are presented as box-and-whisker plots. Data were analyzed by one way ANOVA with Tukey's post hoc test, *$p < 0.05$, **$p < 0.01$, ***$p < 0.001$ compare to the MI group, †††$p < 0.001$ compare to the AH group.

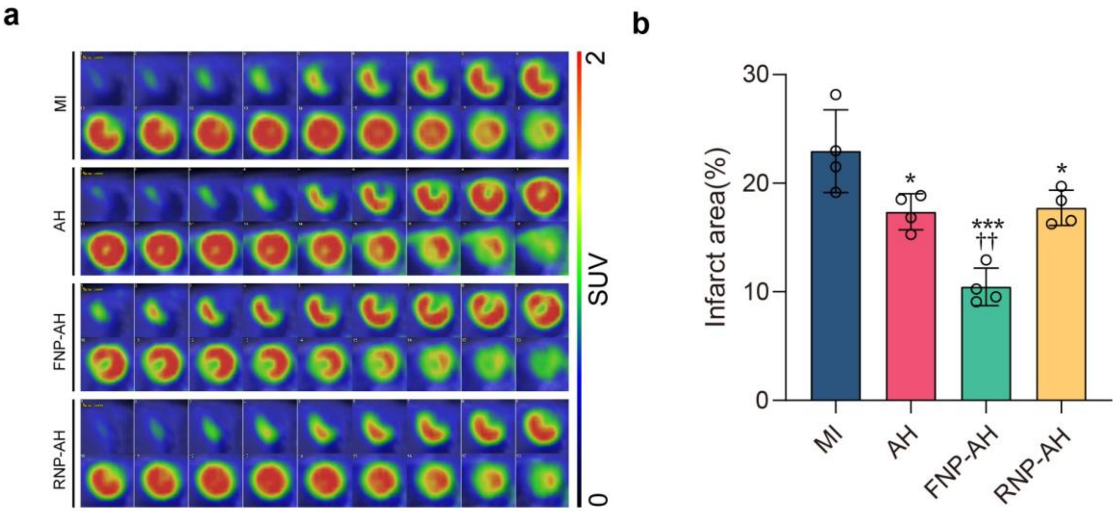

Figure S21. Assessment of infarct area via ^{18}F-FDG PET imaging. (**a**) Representative contiguous PET images acquired at short axis. (**b**) Quantification of infarct size for each group (n=4 biologically independent mice per group). Data are expressed as mean ± s.d. Data were analyzed by one way ANOVA with Tukey's post hoc test, **p* < 0.05, ****p* < 0.001 compare to the MI group, ††*p* < 0.01 compare to the AH group.

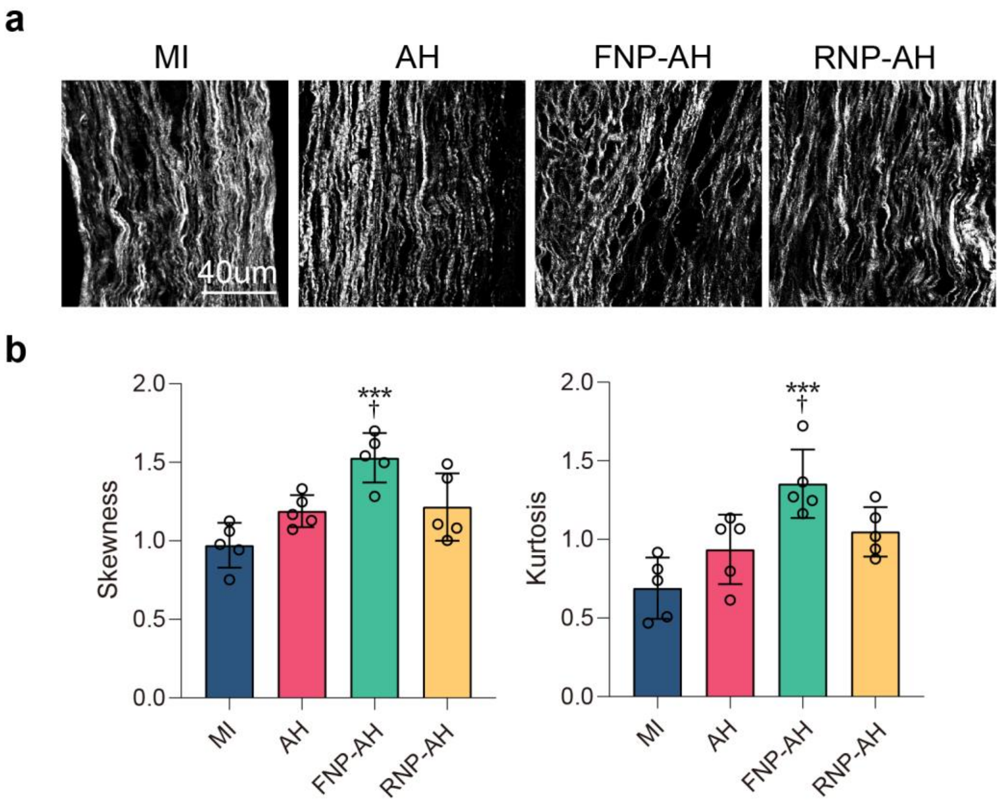

Figure S22. Assessment of collagen fibers morphology via second harmonic generation imaging. (**a**) Representative SHGs images for each group. (**b**) Quantification of skewness and kurtosis as observed in **a** (n=5 biologically independent mice per group). Data are expressed as mean ± s.d. ***p < 0.001 compare to the MI group, †p < 0.05 compare to the AH group.

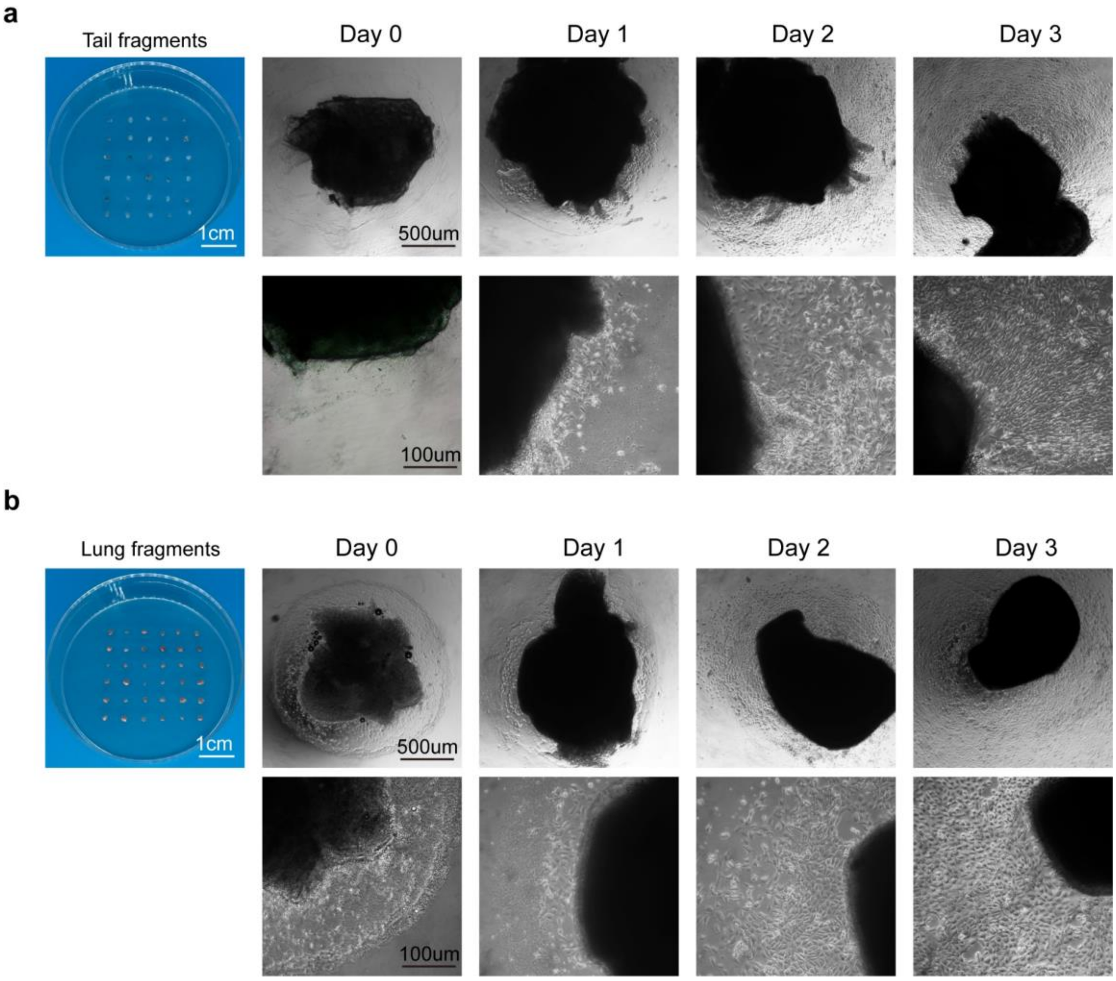

Figure S23. Isolation of skin fibroblasts and lung fibroblasts. (**a**) The tail tip was minced into fragments of approximately 1 mm^2 then placed on 6-cm culture dishes at an interval of ~0.5mm. Skin fibroblasts crawled out of the tail fragment and attach to the dish from day 1. (**b**) The lung was minced into fragments of approximately 1 mm^2 then placed on 6-cm culture dishes at an interval of ~0.5mm. lung fibroblasts crawled out of the tail fragment and attach to the dish from day 1.

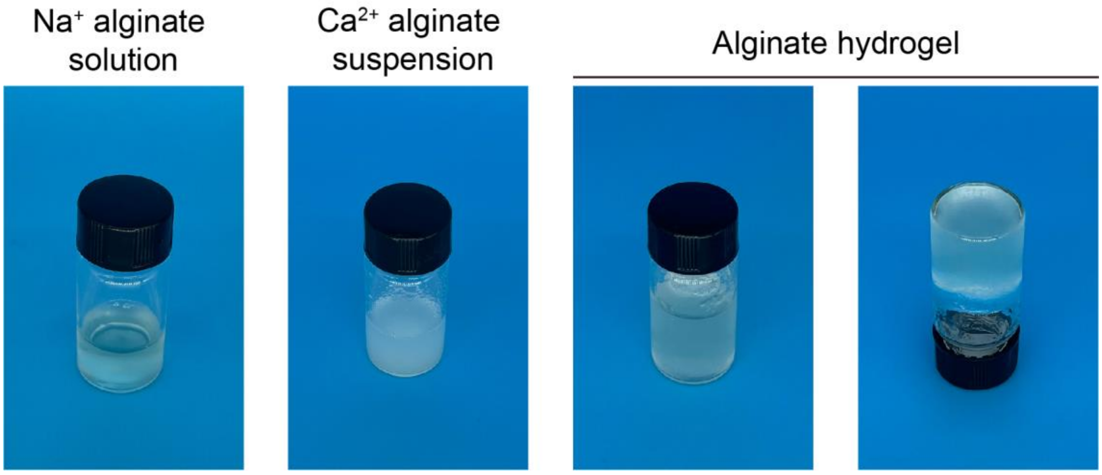

Figure S24. Macrophotograph of Na^{+} alginate solution, Ca^{2+} alginate suspension and alginate hydrogel.

Table S1. Characteristics and quality controls of skin fibroblasts and FNPs.

**Primary mouse skin fibroblasts**	**Target range**
Purity (% of FSP-1^{+} cells)	>95%
Cytokine receptors verification (immunofluorescence)	
TGF-βRII	Positive
IL11RA	Positive
IL13RA	Positive
IL17RA	Positive
Membrane yield	200-300 million cells/ mg protein

**FNPs**	**Target range**
Hydrodynamic size in 1 × PBS (nm)	90-130
PDI in 1 × PBS	< 0.2
Cytokine receptors verification (Western blot)	
TGF-βRII	Positive
IL11RA	Positive
IL13RA	Positive
IL17RA	Positive

Table S2. List of primary/secondary antibodies used in this study.

Primary/secondary antibody	Brand	Art. No.	Lot. No.	application	dilution
S100A4 (FSP-1)	Abcam	ab93283	GR3327654-1	ICC/IHC	1:100
IL-11RA	Abcam	ab125015	GR3356167-5	ICC	1:100
				WB	1:1000
IL-13 receptor alpha 1	Abcam	ab79277	GR213202-17	ICC	1:100
				WB	1:1000
IL-17RA	Abcam	ab180904	GR3304753-5	ICC	1:100
				WB	1:1000
TGF-βRII	Abcam	ab186838	GR3340652-3	ICC	1:100
				WB	1:1000
TGF-β1	Abcam	ab215715	GR3278861-11	IHC	1:500
α-SMA	Proteintech	14395-1-AP	00095164	ICC/IHC	1:150
				WB	1:5000
Collagen-1	Proteintech	14695-1-AP	00080450	IHC	1:500
				WB	1:1000
Collagen-III	Servicebio	GB111629	27717011305	IHC	1:250
E-cadherin	Proteintech	20874-1-AP	00084161	ICC	1:150
				WB	1:10000
GAPDH(HRP-conjugated)	Proteintech	HRP-60004	21002053	WB	1:10000

Fibronectin	Proteintech	15613-1-AP	00080450	WB	1:1000
vWF	Servicebio	GB11020	AC2111067C	IHC	1:400
BrdU	Servicebio	WGB8010	4BbC12	ICC	1:100
Goat Anti-Mouse IgG H&L (Alexa Fluor® 647)	Abcam	ab150119	GR3348378-5	ICC/IHC	1:150
Goat Anti-Rabbit IgG H&L (Alexa Fluor® 488)	Abcam	Ab150077	GR3255194-2	ICC/IHC	1:150
HRP-conjugated Affinipure Goat Anti-Rabbit IgG(H+L)	Proteintech	SA00001-2	20000217	WB	1:5000
HRP-conjugated Affinipure Goat Anti-Mouse IgG(H+L)	Proteintech	SA00001-1	20000216	WB	1:5000

Table S3. Primer sequences used in this study.

Gene	Forward Primer	Reverse Primer
GAPDH	CAGGAGAGTGTTTCCTCGTCC	TTCCCATTCTCGGCCTTGAC
ACTA2	CTTCGTGACTACTGCCGAGC	AGGTGGTTTCGTGGATGCC
COL1A1	CGATGGATTCCCGTTCGAGT	CGATCTCGTTGGATCCCTGG
VIMENTIN	TTTGCTGACCTCTCTGAGGC	CTCCAGGGACTCGTTAGTGC
MMP9	CAAAGGCAGCGTTAGCCAGA	GCGGTACAAGTATGCCTCTGC

Movie S1. Intratracheal administration of FNPs via a microsprayer.

Movie S2. Permanent ligation of the LAD to establish a mouse MI model.

Movie S3. Intramyocardial injection of FNP-AH.

References

[1] A. V. Kroll, R. H. Fang, Y. Jiang, J. Zhou, X. Wei, C. L. Yu, J. Gao, B. T. Luk, D. Dehaini, W. Gao, L. Zhang, Adv. Mater. 2017, 29, 1703969.

[2] J. M. Wang, A. F. Chen, K. Zhang, J. Visualized Exp. 2016, (118), e52965.

[3] J. Sapudom, S. Karaman, W. K. E. Mohamed, A. Garcia-Sabate, B. C. Quartey, J. C. M. Teo, NPJ Regen. Med 2021, 6, 83.

[4] Z. Cao, T. Ye, Y. Sun, G. Ji, K. Shido, Y. Chen, L. Luo, F. Na, X. Li, Z. Huang, J. L. Ko, V. Mittal, L. Qiao, C. Chen, F. J. Martinez, S. Rafii, B. S. Ding, Sci. Transl. Med 2017, 9, eaai8710.

[5] M. Hu, Y. Wang, Z. Liu, Z. Yu, K. Guan, M. Liu, M. Wang, J. Tan, L. Huang, Nat. Nanotechnol. 2021, 16, 466.

[6] a) S. Huang, D. Lei, Q. Yang, Y. Yang, C. Jiang, H. Shi, B. Qian, Q. Long, W. Chen, Y. Chen, L. Zhu, W. Yang, L. Wang, W. Hai, Q. Zhao, Z. You, X. Ye, Nat. Med. 2021, 27, 480; b) J. Tang, T. Su, K. Huang, P. U. Dinh, Z. Wang, A. Vandergriff, M. T. Hensley, J. Cores, T. Allen, T. Li, E. Sproul, E. Mihalko, L. J. Lobo, L. Ruterbories, A. Lynch, A. Brown, T. G. Caranasos, D. Shen, G. A. Stouffer, Z. Gu, J. Zhang, K. Cheng, Nat. Biomed. Eng. 2018, 2, 17.

[7] Q. Fan, R. Tao, H. Zhang, H. Xie, L. Lu, T. Wang, M. Su, J. Hu, Q. Zhang, Q. Chen, Y. Iwakura, W. Shen, R. Zhang, X. Yan, Circulation 2019, 139, 663.

[8] L. Van Hoecke, E. R. Job, X. Saelens, K. Roose, J. Visualized Exp. 2017, (123), 55398.

[9] L. Alonso-Herranz, Á. Sahún-Español, A. Paredes, P. Gonzalo, P. Gkontra, V. Núñez, C. Clemente, M. Cedenilla, M. Villalba-Orero, J. Inserte, D. García-Dorado, A. G. Arroyo, M. Ricote, eLife 2020, 9, 57920.

[10] T. Ashcroft, J. M. Simpson, V. Timbrell, J. Clin. Pathol. 1988, 41 (4), 467.

[11] X. Yan, H. Zhang, Q. Fan, J. Hu, R. Tao, Q. Chen, Y. Iwakura, W. Shen, L. Lu, Q. Zhang, R. Zhang, Circ. Res. 2017, 120, 1116.

[12] S. Anders, P. T. Pyl, W. Huber, Bioinformatics 2015, 31, 166.

[13] T. Wohlfahrt, S. Rauber, S. Uebe, M. Luber, A. Soare, A. Ekici, S. Weber, A. E. Matei, C. W. Chen, C. Maier, E. Karouzakis, H. P. Kiener, E. Pachera, C. Dees, C. Beyer, C. Daniel, K. Gelse, A. E. Kremer, E. Naschberger, M. Sturzl, F. Butter, M. Sticherling, S. Finotto, A. Kreuter, M. H. Kaplan, A. Jungel, S. Gay, S. L. Nutt, D. W. Boykin, G. M. K. Poon, O. Distler, G. Schett, J. H. W. Distler, A. Ramming, Nature 2019, 566, 344.
